# The Assessment of Bioactivity and Biological Responsiveness in Bioactive Glasses and Ceramics: A Review of Available Techniques

**DOI:** 10.3390/ma18184393

**Published:** 2025-09-20

**Authors:** Simone De Micco, Devis Bellucci, Valeria Cannillo

**Affiliations:** Dipartimento di Ingegneria “Enzo Ferrari”, Università degli Studi di Modena e Reggio Emilia, Via P. Vivarelli 10, 41125 Modena, Italy; simone.demicco@unimore.it (S.D.M.); valeria.cannillo@unimore.it (V.C.)

**Keywords:** bioactivity, biological responsiveness, bioactive glasses, ceramics, cell culture assays

## Abstract

The development of bioactive glasses (BGs) and ceramics, such as β-Tricalcium phosphate (β-TCP), Hydroxyapatite (HAp), and apatite-wollastonite (A-W), has revolutionized regenerative medicine (RM), offering innovative solutions for bone and tissue repair, due to the ability of these materials to bond with living bone tissue. Despite significant advancements, evaluating the bioactivity and biological responsiveness of these biomaterials remains a critical challenge. This review provides a comprehensive synthesis of the available methodologies, critically analyzing their advantages, disadvantages, and the possible gap between in vitro and in vivo assessments, including false positives and false negatives. Classical immersion tests techniques for bioactivity evaluation in simulated physiological solutions, such as simulated body fluid (SBF), Tris-buffer (TRIS), or phosphate-buffered saline (PBS) solutions, are discussed, along with the more innovative Simulated Wound Fluid (SWF). Additionally, traditional standardized methods, such as MTT, BrdU, EdU, and XTT, as well as emerging methods like qPCR and immunocytochemistry, used to study cellular behavior, proliferation, adhesion, and differentiation, are compared. Staining assays, including crystal violet, neutral red, and alizarin red, have also been investigated for their effectiveness in evaluating cellular adhesion and quantification. Notably, while all techniques have shown promise in studies involving BGs and ceramics, a multi-parametric approach remains the most reliable strategy for assessing bioactivity and biological responsiveness, highlighting the need for comprehensive studies to validate the results. Finally, the choice between static and dynamic approaches represents a further critical issue, as it significantly affects assay outcomes.

## 1. Introduction

Biomaterials are engineered materials specifically designed to interact with biological systems for medical purposes [1]. Although their traditional field of application has been in prosthetics, in recent years these materials have also been used in regenerative medicine (RM). RM is an interdisciplinary field focused on repairing, replacing, or regenerating damaged or malfunctioning tissues and organs, often utilizing specific biomaterials with tailored properties, stem cells, and molecular biology [2,3]. The goal is pursued through approaches that stimulate self-repair, generate functional tissues, or enhance the body’s natural regenerative capabilities [4].

Generally, the effectiveness of biomaterials relies on their interaction with surrounding tissues, so two fundamental properties must be considered [5], bioactivity and biological responsiveness, both of which can be analyzed through various assays as discussed in the following paragraphs.

Bioactivity is a fundamental property that describes the ability of a biomaterial to interact with biological systems and induce a specific biological response [6]. In the context of biomaterials, bioactivity often refers to the capability to bond with living tissues through the formation of a hydroxyapatite (HAp) layer on the material’s surface, which promotes integration with bone [7,8]. Indeed, HAp closely resembles the mineral component of natural bone, both chemically and structurally [9,10]. Beyond this classical definition, modern perspectives on bioactivity encompass not only the formation of a HAp layer but also dynamic cellular responses—such as proliferation, differentiation, and adhesion—as well as the absence of cytotoxic or genotoxic effects, providing a more comprehensive assessment of biomaterial performance.

In addition to bioactivity, biological responsiveness denotes a biomaterial’s capacity to interact dynamically with cells, tissues, and the immune system. This property includes various aspects, such as the material’s ability to support cell adhesion, proliferation, and differentiation, as well as its influence on signaling mechanisms that regulate tissue regeneration [6].

**Figure 1:**
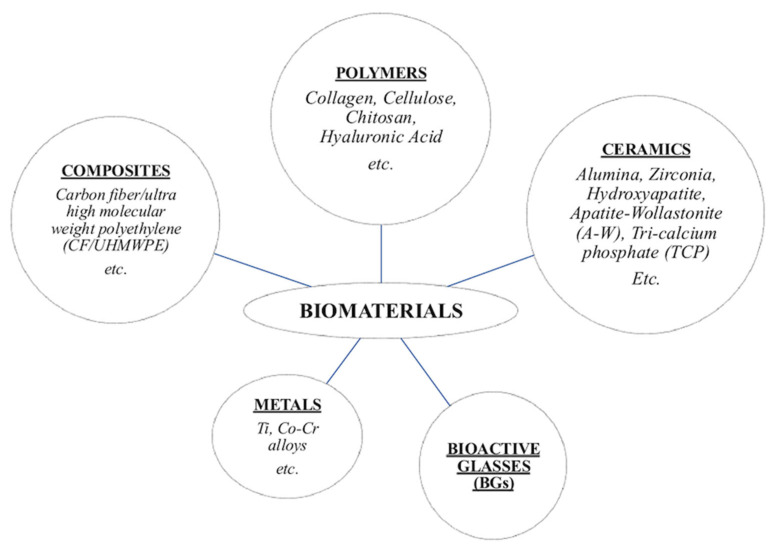
Class of biomaterials used in the human body.

Bioactivity and biological responsiveness are closely related to a material’s composition and structure. Based on their characteristics, biomaterials can be classified into different categories (Figure 1) according to their composition, origin, structure, and function. Chemically, they include metals, polymers, composites, ceramics and bioactive glasses (BGs) [7,8]. Among these, this review will focus on ceramics and BGs, due to their wide application and their relevance in RM. In brief,

(i) Metals are used in load-bearing applications due to their excellent mechanical strength and durability. However, once implanted in the body, they may be susceptible to corrosion;
(ii) Polymers, both natural and synthetic, offer good versatility in terms of degradation rates and processing, making them suitable for soft tissue engineering and drug delivery systems;
(iii) Composites, composed of two or more materials, are engineered to combine the complementary properties of each phase.

Bioceramics are inorganic, biocompatible materials suitable for various biomedical applications [9,10]. They have been extensively studied due to their favorable properties, including high strength and chemical stability [8]. According to their chemical response in physiological environments, bioceramics are classified into three subgroups [11]:

(a) Nearly inert ceramics, which exhibit minimal interaction with biological tissues and are commonly used in joint prostheses and cardiovascular devices;
(b) Surface-reactive ceramics, which actively interact with biological fluids to enhance integration with surrounding tissues;
(c) esorbable ceramics, which, as the name suggests, gradually degrade and are absorbed by the body.

Among bioceramic materials, notable examples include

(i) Tricalcium phosphate (TCP) [12], a calcium salt of phosphoric acid (Ca_3_(PO_4_)_2_), which exists in three polymorphic forms: α-TCP (stable between ~1120 °C and 1465 °C), α′-TCP (stable at temperatures > 1430 °C), and β-TCP (stable at room temperature and transforming into α-TCP at ~1120 °C) [12]. From a biological perspective, both α-TCP and β-TCP are biodegradable and bioactive, with a resorption rate higher than crystalline apatite [13];
(ii) Synthesized apatite, a ceramic similar in composition to bone mineral. The term apatite refers to a group of crystalline compounds including HAp (Ca_10_(PO_4_)_6_(OH)_2_), fluorapatite, and the composite fluoro-hydroxyapatite (FHA) [14]. Synthetic HAp has been widely used in biomedical applications, including porous implants, powders, and coatings for metallic prostheses to enhance bioactive fixation [14]. The incorporation of HAp coatings has been shown to promote bone growth along the implant surface, forming a mechanically strong interface. Additionally, HAp particles can be integrated into polymer matrices such as polyethylene, forming bioactive composites that play a crucial role in bone repair and replacement, particularly in the reconstruction of the middle ear;
(iii) Apatite-wollastonite glass-ceramics (A-W) [15], a bioceramic that creates a bone-like apatite layer on its surface in vivo, enabling strong bonding with surrounding tissues. The A/W glass-ceramic was studied by Prof. Kokubo and colleagues in 1980s and demonstrated excellent bioactivity and mechanical properties, making it a material with great potential for mechanically resilient biomedical applications [16]. Moreover, its composition and microstructure enhance long-term stability in physiological environments [17].

BGs represent another class of biomaterials widely used in tissue engineering (TE) due to their bioactivity, potential resorbability (for example, when used in powder form), osteoconductivity, which refers to their ability to support the attachment and growth of bone-forming cells along the biomaterial surface, facilitating new bone formation [18]. However, their thermal instability can lead to crystallization during heat treatments required for processing or improving mechanical properties. Such crystallization may affect bioactivity and dissolution behavior [19,20]. To highlight the relevance of BGs, several reviews in the literature [21,22,23,24] provide extensive descriptions of their history, properties, and current clinical applications [25].

To better understand the origin and evolution of BGs, a deeper insight into the development of the first BG designed to chemically bond with bone tissue is essential: 45S5 Bioglass^®^. The composition of 45S5, in mol%, is 46.1% SiO_2_, 26.9% CaO, 24.4% Na_2_O, and 2.6% P_2_O_5_, and it was formulated by Prof. L.L. Hench after two years of research beginning in 1967 [26,27]. Hench initially incorporated phosphorus into the glass because it is an essential component of HAp, while sodium was added to decrease the melting point of the glass, facilitating its processing [26]. This careful design of the composition enhanced the material’s reactivity and facilitated its integration with bone, establishing 45S5 as a benchmark for several subsequent BG formulations [28]. Among these, S53P4, commercially known as BonAlive^®^, is a representative example [29], with a composition (mol%) of 53.8% SiO_2_, 21.8% CaO, 22.7% Na_2_O, and 1.7% P_2_O_5_ [30]. Notably, S53P4 possesses intrinsic antibacterial properties, a slow dissolution rate and stable bioactivity.

Two widely used methods are commonly reported in the literature for producing BGs: the melt-quench technique (MQ) and the sol–gel process (SG). MQ technique involves melting oxides and carbonates at high temperatures (>1200 °C), quenching the molten glass in water, and drying it. The resulting material can then be ground to obtain a fine powder or granules, depending on the intended application. If needed, the powder can be further processed through sintering to obtain the desired shape and density [31]. Conversely In contrast, the SG process relies on a transition from a colloidal solution (sol) into a gel, which is then dried and heat-treated to form solid BGs [32,33,34,35,36]. SG-derived BGs generally exhibit higher bioactivity in the early stages of immersion in simulated physiological solutions due to their increased surface area and porosity [37,38,39]. In particular, the porosity of SG-BGs significantly influences their dissolution rate and ion release, which are key factors in their bioactivity and biological responsiveness. Both methods—MQ and SG—achieve similar dissolution and HAp nucleation rates over extended periods [40].

Two widely used methods for producing BGs are the melt-quench (MQ) and sol–gel (SG) processes. In MQ, oxides and carbonates are melted at high temperatures (>1200 °C), quenched in water, and dried; the resulting material can be ground into powders or granules and, if needed, sintered to achieve the desired shape and density [31]. In contrast, the SG process involves the transition of a colloidal solution (sol) into a gel, which is then dried and heat-treated to form solid BG [32,33,34,35,36]. SG-derived BGs generally exhibit higher early-stage bioactivity due to their increased surface area and porosity [37,38,39], which also influence dissolution rates and ion release, key factors in bioactivity and biological responsiveness. Despite these differences, both MQ and SG methods achieve similar long-term dissolution and HAp nucleation rates [40].

Researchers commonly evaluate the bioactivity and biological responsiveness of BGs and ceramic biomaterials using in vitro or in vivo testing methodologies. In vitro assays are conducted under controlled laboratory conditions [41,42] to assess ion release, HAp formation, and cellular interactions using cell cultures or acellular solutions that mimic the ionic composition of human blood plasma. In contrast, in vivo studies involve implantation of biomaterials into living organisms to directly evaluate their performance in a biological environment. These tests provide crucial information on tissue integration, inflammatory response, biodegradation, and long-term stability.

However, in some cases, bioactive materials can exhibit promising behavior in vitro, but their performance in vivo may differ significantly. For instance, certain materials that appear bioactive in vitro may cause toxicity or show limited interaction with living tissue when implanted in vivo, a phenomenon known as a false positive [40]. An example of this is proposed by Xia et al. [43] with nanostructured titanium dioxide (TiO_2_). This material promotes cell proliferation in vitro but may cause inflammatory responses or toxicity in vivo. In contrast, a false negative occurs when a material fails to exhibit a biological effect in vitro, despite showing positive results in vivo [40]. Park et al. [44] proposed an example of a false negative: low-crystallinity HAp, which often shows minimal cell response in vitro but actively promotes bone regeneration and cell proliferation in vivo. In the following sections, specific case studies illustrating these discrepancies will be presented and discussed.

Despite these limitations, the use of in vitro protocols remains a viable alternative for assessing the bioactivity or biological responsiveness of BGs and ceramic biomaterials. This opinion is widely shared by many researchers, as evidenced by the large number of published articles describing in vitro testing.

This review aims to provide a detailed and comprehensive analysis of the main techniques available for assessing the bioactivity or biological responsiveness of biomaterials—with a focus on BGs and various types of ceramics—highlighting their potential and limitations. The discussion will compare different approaches, including static and dynamic assays, as well as direct and indirect contact methods. These comparisons are essential for understanding the suitability of each method for specific applications.

## 2. In Vitro Static and Dynamic Approaches for Evaluating Bioactivity and Biological Responsiveness

In vitro assays can be performed using static or dynamic approaches, each designed to mimic physiological conditions to varying degrees and offering distinct advantages and limitations [45,46] (Table 1). 

Static methods are conducted in a controlled environment, involving evaluation under constant conditions without dynamic parameters such as mechanical stress or fluid flow. These methods focus on immersing the biomaterial in a solution and observing surface changes, ion release, or the formation of a bioactive HAp layer. Static approaches are commonly used due to their simplicity and reproducibility, although they cannot fully simulate the complexity of the biological environment, where cell activity and ion concentrations are constantly changing. As a result, static approaches may provide only a limited understanding of how biomaterials behave in vivo.

In contrast, dynamic tests provide a simplified but more realistic simulation of the physiological environment, introducing the effects of continuous fluid exchange and natural variations in pH and temperature, thus offering a more accurate representation of in vivo conditions. Compared to static tests, dynamic methods more closely simulate the environment of the human body, providing a better basis for evaluating ion release kinetics [49]. However, dynamic approaches are often more difficult to establish and may require more sophisticated equipment and experimental controls. Factors such as fluid flow rate can directly affect sample dissolution [47]. Controlling the flow conditions or monitoring ion concentrations has been proposed to improve reproducibility [47]. In particular,

- Low flow rates (~0.04 mL/min) correspond to high ion concentrations in the solution and a low dissolution rate [47].
- With high flow rates (~0.6 mL/min), dissolution occurs uniformly and is influenced by surface reactions [47].

Static immersion tests are widely used to study bioactivity, providing valuable insights into the initial reactivity of BGs and ceramics. However, they do not account for the continuous renewal of biological fluids that occurs in vivo [50]. To overcome this limitation, dynamic systems such as continuous flow or cascade reactors have been introduced [50]. For example, Siekkinen et al. [51] investigated the dissolution behavior of 45S5 Bioglass^®^ in a cascade reactor to better mimic in vivo conditions. Dissolution kinetics were evaluated using Tris-buffer (TRIS) and simulated body fluid (SBF), which differed in ionic composition and buffering capacity. TRIS allows controlled studies of fundamental dissolution mechanisms, whereas SBF better simulates the physiological environment (a detailed comparison of these solutions is discussed later). Under static conditions, ions accumulate, leading to rapid solution saturation, slowed BG dissolution, and limited HAp formation. In contrast, dynamic systems promote faster ion release and HAp formation, particularly in the initial stages of flow.

Dynamic approaches are increasingly being explored in biological responsiveness studies. Bioreactors, which enable precise control over fluid flow, oxygenation, and mechanical stimuli, are widely used to enhance cell proliferation, differentiation, and extracellular matrix deposition. More recently, organ-on-a-chip (OOC) [52] systems have emerged as an innovative tool to replicate physiological microenvironments in vitro. These microfluidic platforms integrate controlled fluid flow with 3D cell cultures, more accurately mimicking tissue-specific conditions than traditional static cultures. Syahruddin et al. [52] compared different OOC setups for studying bone tissue engineering applications in dentistry, demonstrating their potential to model complex cell-material interactions under dynamic conditions. Although OOC technology is still in its early stages, it represents a promising alternative for evaluating the biological response of bioactive materials.

Overall, both static and dynamic approaches have been used to study bioactivity, whereas in the assessment of biological responsiveness, static methods remain the most widely adopted due to their simplicity and their ability to minimize experimental complications. Static conditions can lead to progressive pH changes caused by ion accumulation, which in turn affects HAp formation by altering the supersaturation of Ca^2+^ and phosphate ions. In contrast, dynamic systems maintain more stable pH values by facilitating buffering control and removing dissolution products, providing a closer approximation of in vivo conditions.

## 3. In Vitro Assays for the Study of Bioactivity

**Figure 2:**
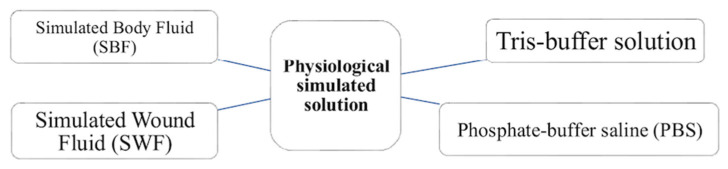
Physiological simulated solutions to test bioactivity in vitro.

As previously mentioned, bioactive materials typically induce bonding with bone tissue through HAp layer precipitation [53,54]. This process can be reproduced in vitro using simulated physiological solutions [55] (see Figure 2) and is applicable to both BGs and ceramics. BGs, being amorphous and highly reactive, release ions rapidly, promoting supersaturation and HAp formation. In contrast, crystalline ceramics require more time, often resulting in HAp layers that are less uniform in structure.

### 3.1. Simulated Body Fluid (SBF)

SBF is one of the most widely used physiological solutions for evaluating the bioactivity of BGs and ceramics. It is standardized for bulk materials according to ISO/FDIS 23317:2007(E) [56], which recommends the use of disk or rectangular plate specimens. This standard relies on the principle that bioactivity can be evaluated by the creation of a HAp layer on the material’s surface after immersion in the solution. Specific, commonly accepted guidelines also exist for testing BG powders in SBF [57]. The use of this testing assay was originally introduced by Kokubo et al., who suggested reproducing in vitro the precipitation of HAp on bioactive materials and proposed the SBF solution, which has been gradually refined over time [40]. These refinements culminated in the 2006 formulation [53], known as the original SBF (see Table 2).

SBF is an acellular inorganic solution with a composition similar to human blood plasma, but it lacks organic components [53]. Compared to plasma, it contains higher levels of chloride (Cl^−^), calcium, and phosphorous ions [40], and lower bicarbonate (HCO_3_^−^) levels [58]. 

To prepare the SBF solution according to the original protocol developed by Kokubo et al. [53], the following reagents are sequentially dissolved in distilled water at 36.5 °C: sodium chloride (NaCl), sodium hydrogen carbonate (NaHCO_3_), potassium chloride (KCl), magnesium chloride hexahydrate (MgCl_2_∙6H_2_O), hydrochloric acid solution 1 M (HCl), calcium chloride (CaCl_2_), sodium sulfate (Na_2_SO_4_), and tris-hydroxymethyl aminomethane TRIS ((HOCH_2_)_3_CNH_2_), and di-potassium hydrogen phosphate trihydrate (K_2_HPO_4_∙3H_2_O). The solution remains colorless, transparent, and free of precipitates during preparation. During immersion, the solution is periodically replaced to ensure that fresh reactive ions are available for interaction with the samples [59]. Then, the samples should be dried, and the surface analyzed by various techniques [60,61], such as X-Ray diffraction (XRD), Fourier-transform infrared (FT-IR) or Raman spectroscopy [62], and scanning electron microscopy (SEM) [63], to monitor the formation of the HAp layer [55,64,65].

However, although the formulation by Kokubo et al. remains widely adopted, researchers have explored alternative ionic concentrations to better replicate the physiological environment:

(i) Kim et al. [66] demonstrated that the HAp layer formed in SBF differs from bone apatite in both composition and structure, attributing these differences to SBF’s higher Cl^−^ content and lower HCO_3_^−^ concentration compared to blood plasma. Based on these findings, Oyane et al. [67] revised the original SBF and introduced new variants (c-SBF, r-SBF, i-SBF, and m-SBF) with ion concentrations more closely matching those of blood plasma (Table 3) and supersaturated with respect to HAp. The results demonstrated that upon storage, r-SBF and i-SBF showed no changes in ion concentrations but exhibited a decrease in HCO_3_^−^ and an increase in pH. In contrast, c-SBF and m-SBF maintained stable ion concentrations for a longer period, indicating that r-SBF and i-SBF are less stable in terms of ionic composition. Among these formulations, m-SBF was found to have the most optimal ion composition for assessing bioactivity in vitro and for the biomimetic synthesis of HAp.

(ii) Müller et al. [68] explored the role of HCO_3_^−^ by proposing an SBF formulation with varying bicarbonate concentrations, as these ions affect the chemical composition and the structure of the calcium phosphate that form. At low HCO_3_^−^ concentrations, only carbonate hydroxyapatite (HCA), in which carbonate ions replace phosphate groups, is formed. Conversely, at higher bicarbonate concentrations, HCA crystals in which carbonate ions substitute hydroxyl groups are synthesized.
(iii) Mei et al. [69] investigated the influence of proteins on the reaction kinetics of BGs in modified SBF solutions. In their study, proteins such as bovine serum albumin were added to better mimic the biological environment. The presence of proteins affected the surface reactions of BGs in vitro, modifying the dissolution rate as well as the composition and structural organization of the layer formed on the material’s surface.

As part of simulating physiological conditions, the pH of SBF is maintained at approximately 7.4 by using Tris (NH_2_C(CH_2_OH)_3_), but it tends to shift toward alkaline values [58] due to ion exchange at the material’s surface. Specifically, samples release alkali ions into the solution in exchange for H^+^ ions. Additionally, the dissolution of the silicate network contributes to the release of basic compounds, further increasing alkalinity. This rise in solution pH plays a critical role in HAp nucleation, making it particularly important to buffer the solution.

The study conducted by Pan et al. [70] demonstrated differences in the HAp-forming ability of silicon-based bioactive materials in SBF, including CaSiO_3_ (wollastonite), 45S5 Bioglass^®^, and borate glasses. Their results indicated that the HAp crystal morphology is influenced by pH, with spherical aggregates of plate-like crystals forming on 45S5, rod-like structures on CaSiO3, and a porous multilayer coating on borate glass. These findings demonstrate that pH and material degradation affect HAp formation mechanism and morphology. Attempts to substitute Tris with alternative buffers, such as Good’s buffers like HEPES (4-(2-hydroxyethyl)piperazine)-1-ethanesulfonic acid) [71] and MOPS (3-[N-morpholino] propanesulfonic acid) [72], have been unsuccessful in stabilizing the pH.

Additionally, the solution pH can also impact cell viability [70]. In the case of borate glass, its rapid ion release often leads to an increase in pH, which can negatively affect osteoblast adhesion and morphology, as observed by SEM. For example, in the cited study [70], starting from the third day of immersion, cell contraction was evident, with precipitated HAp partially covering the cell surfaces. By day 7, no viable cells remained, and the surface was completely covered by a thick HAp layer.

Overall, SBF testing is not always a definitive predictor of in vivo bioactivity, as demonstrated by several studies. Additionally, the widely used Kokubo and Takadama protocol for preparing SBF has several limitations, including its complexity, lack of filtration, and uncontrolled carbonate content [53]. Based on the leading opinion paper by Kokubo and Takadama [53], which is founded on the premise that “a material able to have apatite form on its surface in SBF has apatite produced on its surface in the living body, and bonds to living bone through this apatite layer”, Bohner et Lemaitre [73]

(i) presented several examples that contradict this assumption, including BGs, β-TCP, HAp, dicalcium phosphate dihydrate (DCPD), and calcium sulfate hemihydrate (CSH). Discrepancies in SBF testing arise from material solubility and local changes in supersaturation. Highly reactive BGs increase local pH, reducing HAp solubility and accelerating apatite nucleation, potentially overestimating in vitro bioactivity. Similarly, DCPD or CSH release high amounts of calcium and phosphate ions, inducing rapid HAp precipitation. However, they dissolve too quickly in vivo to establish a stable bond with bone, resulting in false positives.
(ii) showed that the selection of an SBF solution is often arbitrary and proposed an iterative method to refine the in vitro approach. This involves selecting a simple SBF that mimics blood serum properties, testing reference materials, and investigating the influence of additives to improve the correlation between in vitro and in vivo results, reducing the risk of false positives or false negatives.

Another critical aspect of SBF in vitro analysis is the testing mode. Static conditions are typically preferred for bulk samples, such as disks, while dynamic conditions are more appropriate for fine powders or particulates [74]. In this context,

(i) Zhang et al. [75] investigated the effect of fluid circulation on the in vitro bioactivity of 45S5 Bioglass^®^ granules (500–800 μm), demonstrating that dynamic conditions significantly influence HAp formation. Under static conditions, reaction layers were thicker, often presenting a distinct calcium-phosphate coating on the top of the silica gel layer. Conversely, under dynamic conditions, the reaction deposits were thinner and more homogeneous, with calcium-phosphate inclusions appearing as a single mixed layer. HAp formation under static conditions depended on the position of the samples, whereas under dynamic conditions, a uniform layer formed on all particles.
(ii) Similarly, Ràmila et al. [76] conducted a comparative study on the in vitro bioactivity of a sol–gel-derived SiO_2_-CaO-P_2_O_5_ glass, evaluating its behavior in static and dynamic SBF conditions. Under dynamic conditions, Ca^2+^ concentration and pH remained stable and comparable to those of human blood plasma, making this approach more suitable for predicting in vivo bioactivity.
(iii) Following the same approach, Kang et al. [77] examined the in vitro bioactivity of a poly(L-lactic acid)/β-TCP porous scaffold. Their results show that fluid circulation significantly reduced the degradation rate of molecular weight and compressive strength.

These findings indicate that static SBF tests tend to promote thicker and more localized reaction layers, while dynamic SBF tests result in thinner and more uniform layers. Moreover, dynamic conditions help maintain stable ion concentrations and pH, providing a more predictive assessment of bioactivity.

Several studies have demonstrated the versatility and broad applicability of the SBF assay with BGs and ceramics. Among these, the work by Baino et al. [74] represents a comprehensive analysis of the evolution of in vitro bioactivity testing methods using SBF, highlighting the influence of solution formulation and circulation, as well as material composition and geometry. The authors compared the results obtained with sintered HAp, β-TCP, and A-W glass-ceramics. Notably, β-TCP can bond to bone without necessarily forming an HAp layer, highlighting differences in bioactive behavior compared to other ceramics.

BGs are highly versatile for applications involving both bone and soft tissue integration; however, they are also highly sensitive to testing conditions, such as the mass-to-volume ratio of SBF (Table 4), which may lead to unwanted calcium carbonate precipitation. CaCO_3_ precipitation is promoted by high solid-to-liquid ratios, pH drift, and high HCO_3_^−^ content. To mitigate this effect, the literature suggests: (i) considering revised SBF variants to stabilize pH and carbonate; and (ii) regularly replacing SBF and verify pH (7.40 ± 0.02) at 37 °C [74]. Conversely, ceramics such as β-TCP are chemically more stable and less reactive, exhibiting a type of bioactivity that does not always depend on HAp formation.

To conclude, the SBF method is a simple and inexpensive approach to qualitatively evaluate the bone-bonding capacity of a biomaterial [79]. However, the results are strongly influenced by several factors, such as the composition of SBF and its supersaturation with respect to HAp [80]. The immersion test can also affect certain biomaterial properties, including ion release kinetics and water affinity [81]. Finally, it should be noted that, in SBF, HAp formation can occur directly from the solution, whereas in other fluids this process requires the dissolution of the biomaterial, followed by the subsequent deposition of elements such as calcium and phosphorus, which will then constitute the HAp layer [82,83].

### 3.2. Tris-Buffer Solution (TRIS)

The TRIS-buffer (tris(hydroxymethyl)aminomethane) (TRIS) method is a simpler simulated physiological solution used to evaluate the bioactivity of BGs [82,84] and ceramic biomaterials [85].

**Figure 3:**
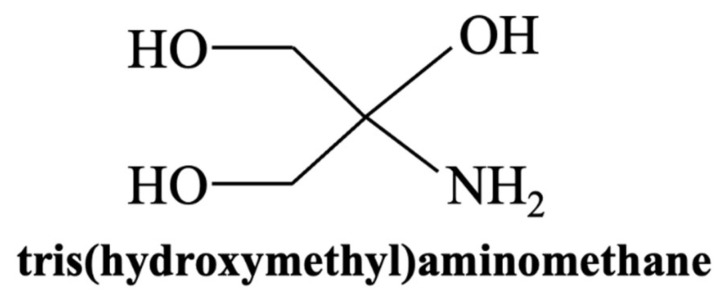
Chemical structure of TRIS (tris(hydroxymethyl)aminomethane).

TRIS is commonly prepared by dissolving tris(hydroxymethyl)aminomethane (Figure 3) in deionized water and adjusting the pH to 7.4 using hydrochloric acid (Tris-HCl) [82] (Table 5). Alternative formulations, including acetic-acid-based (Tris-HAc [86]) or lactic-acid-based (Tris-LA [87]) buffers, avoid the presence of chloride ions. These modified TRIS solutions, which do not contain high concentrations of ions associated with HAp formation, are particularly useful for studying the early stages of ion release [86,88] and the initial phases of HAp formation [86]. As in SBF protocol, this method involves immersing the sample in the solution, typically maintained at 37 °C to replicate human body conditions [89].

Unlike the more complex SBF, TRIS contains no Ca, P, or other ions prone to spontaneous precipitation. This makes it particularly useful for studying BG dissolution and reprecipitation without interference from external ions [90]. In other words, in TRIS, HAp formation depends solely on ion release from the biomaterial. Bellucci et al. [91] compared the in vitro reactivity of various Na_2_O/K_2_O-CaO-P_2_O_5_-SiO_2_ BGs in both SBF and TRIS, confirming that TRIS is a valuable alternative to SBF, especially when a slower reaction rate is desirable to monitor sample evolution over time. The dissolution process in TRIS-buffer is crucial, as it promotes the release of essential ions such as Ca^2+^ and PO_4_^2−^ from the bioactive material [88,91,92,93]. However, compared to SBF, the development of the HAp coating proceeds at a slower rate [88].

Several studies have explored the impact of TRIS on surface reactivity of BGs and glass-ceramics. Following the approach of Mei et al. [69], Radin et al. [94] investigated the progressive modifications of the BG surface in vitro under increasingly complex conditions, starting with TRIS solution and sequentially incorporating serum proteins, plasma electrolytes, and bone cells (osteoblasts). The gradual introduction of biological components influenced the composition, structure, and morphology of the surface reaction layer. The findings revealed the formation of two distinct layers on the material surface: a silica-gel deposit and a secondary layer composed of Si intermixed with amorphous Ca–P phases.

Additionally, Rohanová et al. [92] and Hlvác et al. [93] examined the dissolution behavior of glass-ceramics in TRIS. Rohanová et al. focused on a glass-ceramic scaffold derived from 45S5 Bioglass^®^, consisting of 77 wt.% crystalline phases (Na_2_O·2CaO·3SiO_2_ and CaO·SiO_2_) and 23 wt.% of residual glass phase. In contrast, Hlvác et al. investigated an A-W type glass-ceramic. The results from both studies indicate that TRIS forms soluble complexes with Ca^2+^ ions, affecting the deposition of the HAp layer on the material surface. This layer typically acts as a diffusion barrier, influencing the release kinetics of other ions. Both studies demonstrated that the formation of Ca^2+^-TRIS complexes continuously removes free calcium ions from the solution, preventing their accumulation and sustaining the dissolution process over time. This has important interpretative consequences, since the continuous removal of free Ca^2+^ ions inhibits HAp nucleation and may lead to an underestimation of the bioactivity. Practical workarounds include combining TRIS tests with complementary analyses, such as ion release monitoring or parallel assays in SBF.

Different studies in the literature have also reported the use of a dynamic setup with TRIS. In particular, the study conducted by Fagerlund et al. [95] is relevant because, by applying a dynamic flow setup, the authors investigated the dissolution behavior of BGs with varying compositions in TRIS. Their results suggest that monitoring initial ion dissolution under dynamic conditions can serve as an effective pre-screening approach for evaluating the suitability of different BG compositions. Additionally, the ability of BGs to form an HAp layer and bond to bone was found to depend on the concentrations of calcium and silicon released from the material itself.

In conclusion, the TRIS-buffer method is particularly useful for evaluating the initial stages of bioactivity, focusing on dissolution and reprecipitation processes that are fundamental for biomaterial integration with bone tissue. Unlike SBF, TRIS does not replicate the complete ionic composition of physiological fluids, making it less suitable for simulating the later stages of biomineralization. In TRIS, BGs typically exhibit faster ion exchange and HAp formation compared to ceramics like sintered HAp or β-TCP, which dissolve at a slower rate. Despite its limitations, TRIS remains a key tool for fundamental in vitro investigations on the dissolution behavior of biomaterials.

### 3.3. Phosphate-Buffer Saline (PBS) Solution

Phosphate-buffer saline (PBS) is widely employed as an alternative in vitro solution to investigate the bioactivity, dissolution behavior, and surface reactivity of biomaterials under conditions that mimic the human body [96].

PBS is a water-based saline solution with a pH of about 7.4 and a composition close to human blood plasma [55] (Table 6). It contains NaCl, Na_3_PO_4_, and K_3_PO_4_, but it lacks essential ions such as K^+^, Mg^2+^, Ca^2+^, HCO_3_^−^ and SO_4_^2−^ [55]. Additionally, PBS contains a twentyfold higher HPO_4_^2−^ concentration than human blood [97], which may influence the dissolution kinetics and bioactivity response of biomaterials.

In a typical PBS immersion test, samples are immersed at 37 °C to reproduce in vivo conditions. This method mainly aims to study the dissolution process of the biomaterial and the release of bioactive ions, including calcium (Ca^2+^), phosphate (PO_4_^2−^), and silicon, which are essential for bone bonding and tissue regeneration. The rapid leaching of ions from samples may cause a significant rise in the pH of the PBS solution, promoting HAp nucleation on the sample surface [97]. Key parameters such as sample weight loss and pH changes are often monitored to evaluate degradation behavior [64].

Several studies have investigated the dissolution behavior of BGs and composite biomaterials in PBS. For example,

(i) Yusof et al. [55] explored the impact of incorporating B_2_O_3_ into 45S5 Bioglass^®^, emphasizing the importance of understanding element dissolution from BGs in PBS, as this correlates with the material’s bone-bonding ability in vivo. Their study proposed an Equation (1), based on prior research [98], to determine the dissolution rate of each element from the sample immersed in the physiological solution [55]:

(1) $$D_{R}\left(\frac{ppm}{day}\right)=\left(\frac{C_{i}-C_{0}}{C_{S}}\right)\cdot \frac{1}{T}$$

where *C_i_* is the concentration of the element obtained from ICP-OES, *C*_0_ is the background concentration, *C_s_* is the concentration of the respective element in the solution, and *T* is the time of immersion in solution. Their findings confirmed that a high Si concentration indicates a rapid glass dissolution [55]. However, the proposed equation is not normalized to sample surface area or mass, which may complicate direct comparisons across different specimen geometries. This limitation highlights the need for future studies to adopt surface area- or mass-normalized metrics to improve comparability.

(ii) Zhou et al. [99] investigated the in vitro degradation behavior of BG-polymer composites in PBS, demonstrating that the incorporation of BG particles altered the dissolution kinetics of the polymer. The acidic degradation products of the polymeric matrix interacted with PBS and were neutralized by the alkaline degradation products released from the BG particles.
(iii) Loh et al. [97] studied calcium-based 45S5 BG pellets in PBS, reporting the formation of carbonate apatite, fluorapatite, and calcite phases. Their study highlighted that the interaction between BGs and PBS accelerates over time, leading to progressive HAp development on the material’s surface.

Overall, PBS represents a suitable in vitro soaking solution for its stability in long-term studies, where the gradual increase in Ca and P content suggests the potential formation of an HAp layer on the biomaterial surface [97]. However, in several studies, PBS is employed in combination with other physiological simulated solutions or analytical techniques to ensure a more accurate evaluation of bioactivity.

Additionally,

(a) Most studies have been conducted with static immersion in PBS, while dynamic testing in this medium has not yet been investigated in the literature; dynamic approaches are more commonly reported with other buffered solutions, such as SBF or TRIS;
(b) Cases of false positives or false negatives in PBS have received limited attention in the literature, even though Bohner et al. [73] conducted similar tests as those reported for SBF, and their conclusions were consistent with previous findings.

These gaps in research limit the understanding of biomaterial interactions in flow environments, and future studies should explore dynamic PBS testing to better simulate physiological conditions.

### 3.4. Simulated Wound Fluid (SWF)

In recent years, Simulated Wound Fluid (SWF) has been explored as a possible option for studying the bioactivity of biomaterials used for soft tissue repair or wound healing applications [100], since its composition aims to replicate some aspects of the biochemical environment of wounds. The SWF is prepared by dissolving NaCl, KCl, NaHCO_3_, and NaH_2_PO_4_ in distilled water. The pH is adjusted to 7.4, and the solution is used to assess bioactivity by monitoring HAp precipitation.

Mehrabi et al. [101] studied, for the first time, the apatite-forming ability of SiO_2_-CaO-B_2_O_3_-ZnO glass powder in SWF instead of conventional SBF. Following the standard in vitro bioactivity procedure used with SBF, each glass powder is immersed in SWF using a specific ratio: 75 mg of BG per 50 mL of simulated fluid. HAp formation was then evaluated using XRD, Raman, or FT-IR spectroscopy. The results showed the formation of cauliflower-like precipitates—characteristic aggregates of HAp with a globular and porous morphology resembling the surface of a cauliflower. HAp precipitated on the surface of the glass after immersion in SWF for 14 days.

Using the same approach, Lusvardi et al. [100] evaluated mesoporous BGs-containing hydrogels doped with cerium and additionally loaded with polyphenols in SWF, targeting chronic wound healing. Hydrogels are polymeric materials that can absorb large amounts of water without losing structural integrity [102]. The study in SWF revealed that these hydrogels can release ions, which help stimulate cell activity and supports angiogenesis, i.e., the formation of new blood vessels from pre-existing ones.

In conclusion, SWF is not supported by standardized testing protocols, unlike SBF, but it can be considered a viable alternative, although only a limited number of studies in the literature have reported its use. The presence of NaHCO_3_ and NaH_2_PO_4_ in SWF contributes to pH buffering and ion exchange, potentially influencing the mechanisms of mineral deposition and bioactivity. Currently, the literature lacks studies on SWF application for evaluating the bioactivity of ceramic materials under dynamic conditions. Moreover, no data are available on the possibility of false positive or false negative results.

### 3.5. Comparative Analysis of Simulated Physiological Solutions for In Vitro Bioactivity Assessment

Selecting an appropriate immersion solution is a key factor in evaluating the in vitro bioactivity of BGs and ceramics. Each simulated physiological solution (SBF, TRIS, PBS, and the more recent SWF) provides a distinct chemical environment that influences ion exchange, surface reactivity, and HAp formation. The comparison presented in Table 7 highlights their respective advantages and limitations based on the intended application and test duration.

Beyond solution composition, material-specific properties such as surface area, geometry, and surface charge play key roles in dissolution and precipitation dynamics [104]. Test conditions (static vs. dynamic) and parameters like material-to-medium volume ratio also significantly affect the results. While ISO 23317:2014 recommends a specific surface area-to-volume ratios for static tests (Volume = 100 × Area), more recent approaches suggest fixed mass-to-volume ratios with agitation to enhance test reproducibility (as reported in Table 4).

Several studies reviewed the differences between simulated physiological solutions. For example, Wetzel et al. [88] compared the HAp formation into SBF and TRIS on 45S5 Bioglass^®^ and two new BG compositions containing low concentrations of zinc or magnesium. When immersed in TRIS, phosphate is often the limiting element for HAp formation. This frequently results in the formation of other crystalline phases, such as carbonates. At the same time, the higher phosphate concentration in SBF compared to TRIS facilitates HAp formation in SBF. However, the presence of Mg^2+^ and Zn2+ ions—both of which have a smaller ionic radius than Ca^2+^—can interfere with HAp crystallization. These ions tend to incorporate into the crystal lattice or adsorb onto the surface of nuclei, disrupting the orderly formation of HAp. As a result, their presence slows the nucleation and growth of HAp in SBF, particularly in formulations containing Mg^2+^.

## 4. Cell Culture Assays for the Study of Biological Responsiveness

Assessing biological responsiveness is fundamental for characterizing new biomaterial compositions [105]. A wide range of testing methods [6,106] is available to investigate key cellular behaviors [107]. For this purpose, different cell types derived from distinct tissues and organisms are utilized [6]. In particular, the following tests can be mentioned:

(i) *Cytotoxicity tests* [54]: Used as a preliminary method to assess the safety of a biomaterial. These tests evaluate cell lysis (cell death), inhibition of cell growth, and the ability of cells to survive and proliferate after exposure to potentially toxic materials or substances (e.g., colony formation assays). Additional cellular effects induced by the biomaterial may also be considered. ISO 10993-5 recommends three methods for cytotoxicity testing: extract dilution, direct contact, and indirect contact methods. These are described in detail in the following paragraphs.
(ii) *Hemocompatibility tests* [108]: Used to evaluate the effects of biomaterials when in contact with blood. A common example is haemolysis test, which determines the extent of red blood cell lysis and the release of hemoglobin induced by the biomaterials.
(iii) *Genotoxicity tests* [107]: Used to detect gene mutations and changes in chromosome structure or number caused by biomaterials.
(iv) *Sensitization test:* Used to estimate the potential of a biomaterial to cause contact sensitization.
(v) *Irritation test:* Used to evaluate the irritation potential of materials when applied to a specific site. The tests are conducted in accordance with ISO 10993-1 and ISO 10993-2.
(vi) *Scratch test* [109]: A simple and cost-effective method for measuring cell migration. It involves creating a “scratch” in a cell monolayer and capturing microscopic images immediately afterward and at regular intervals during incubation to monitor cell movement. Compared to other cell culture assays, the scratch test typically requires a longer duration and a larger quantity of cells and reagents.

These methods are both sensitive and accurate and must be conducted in compliance with good laboratory practices. The results should be both reproducible and repeatable. Although numerous in vitro cell culture tests are available, only a few standardized approaches exist for evaluating biomaterials [6], as outlined in detail in the ISO 10993 standards [110,111].

## 5. Methods for In Vitro Biological Cellular Assays

Cytotoxicity tests employ tissue cells in vitro to evaluate cell growth, reproduction, and adhesion to the surface of tested materials, in accordance with International Standards ISO 10993: (i) 10993-1 for the selection of tests; (ii) 10993-12 for sample preparation and reference materials; (iii) 10993-5 for the in vitro methods [110,111]. ISO 10993-5 describes three approaches for cellular biological testing of cytotoxicity, each with distinct advantages and disadvantages (Table 8) [79,112]: tests on extracts, direct contact, and indirect contact methods, including the *agar diffusion assay* and *molecular filtration* [113].

*Extract tests*, sometimes ambiguously referred to as *indirect tests* in the literature—a term also used for indirect contact tests—are used to evaluate the presence of leachable substances released from a material when exposed to a biological medium. These tests involve incubating the material in a solvent under controlled conditions, followed by exposing cell cultures to the extracted solution to evaluate potential cytotoxic effects [114]. The extraction process must be performed under sterile and aseptic conditions to ensure the validity of the results and to maintain statistical reliability; at least three replicates are required for both test samples and controls. The concentration of substances in the extract depends on various factors, including the sample surface area-to-solution volume ratio, pH of the biological medium, environmental temperature, and extraction duration [114].

*Direct methods* examine the immediate interaction between test materials and biological systems. Cells are cultured directly on the material and incubated under appropriate conditions. This approach provides real-time information on cell adhesion, proliferation, and interaction with the material, offering a comprehensive understanding of its biocompatibility. The test is typically conducted by observing changes in cell morphology and number. However, it requires a complex experimental setup and may not be suitable for materials with irregular shapes or rapid degradation rates.

*Indirect contact methods* evaluate the biological response to potentially toxic substances released from a material, without direct exposure of the cells to the solid sample. In these tests, the material is separated from the cell culture by a barrier, such as an agar layer or a diffusion filter. The agar diffusion assay is suitable for biomaterials with high toxicity and limited permeability. In contrast, diffusion filter assays are appropriate for evaluating the biocompatibility of small molecular weight toxic components. Indirect contact methods are especially useful for materials that may interfere with direct contact tests due to their shape, degradation behavior, or specific surface properties. These methods are commonly used as preliminary screening tools because they are simple to perform.

**Figure 4:**
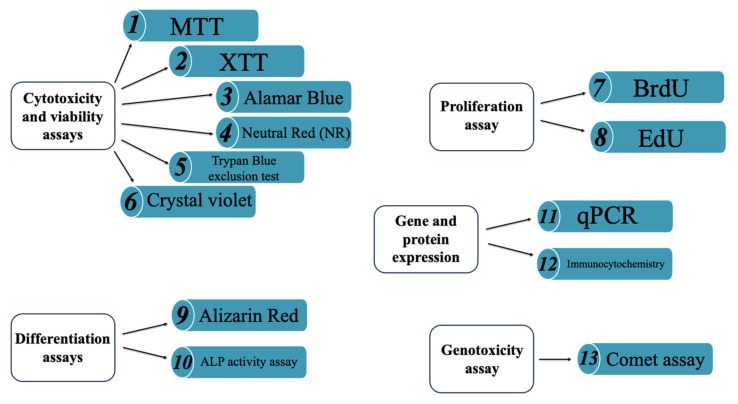
Some examples of techniques utilized to evaluate biological responsiveness. (1–6) cyto-toxicity and viability assays; (7) proliferation assay; (8–9) differentiation assays; (10–12) gene and protein expression assays; (13) genotoxicity assay.

As shown in the studies discussed in the following sections (Figure 4), working with material extracts is the most commonly adopted approach, as it minimizes complications associated with surface properties such as roughness and topography, which must be carefully considered in direct contact assays.

For a reliable assessment of the biological responsiveness of BGs and ceramics, it is essential to integrate results from multiple methods. The combination of approaches, known as a multi-parametrical approach, involves the use of complementary assays to assess various aspects of cell behavior, thereby reducing the risk of false positives or negatives [79,114,115]. Among these assays, viability tests based on membrane integrity, such as dye exclusion assays using Trypan Blue or Crystal Violet, are commonly employed. These methods are based on the principle that viable cells with intact membranes are impermeable to dyes, whereas non-viable cells allow dye penetration. However, dye uptake is often evaluated visually, which can lead to subjective interpretations, particularly when compared to fluorescence-based tests. The latter are more sensitive but also more labor-intensive [116].

## 6. Cell Type Selection for In Vitro Biological Evaluation

The choice of cell type is a critical factor in the in vitro evaluation of BGs and ceramic biomaterials, as it significantly influences the reliability and biological relevance of the results. Different types of assays require specific cell lines, depending on the intended biological outcome (Table 9):

(i) For cytotoxicity tests, the standardized protocol ISO 10993-5 [111] recommends the use of: (a) L-929 fibroblast cells for MTT and XTT assays, due to their high sensitivity to toxic substances and reproducible growth characteristics; (b) BALB3T3 fibroblasts for Neutral Red (NR) assay, because of their rapid proliferation, which ensures consistent and comparable results. Although not formally included in ISO 10993-5, the Trypan Blue exclusion assay is frequently used with L-929 fibroblasts, which are specified in the standard protocol.
(ii) For proliferation assays (e.g., BrdU or EdU assays), human mesenchymal stem cells (hMSCs) and pre-osteoblasts such as MC3T3-E1 are commonly used due to their relevance in bone regeneration studies.
(iii) In osteogenic differentiation assays, including alkaline phosphatase activity (ALP) test and Alizarin red staining, hMSCs are widely used because of their multipotency, while MC3T3-E1 and Saos-2 osteoblast-like cells serve as established models for studying osteoblastic behavior.
(iv) Gene and protein expression studies (e.g., qPCR, immunocytochemistry) similarly rely on osteogenic, pre-osteoblast, or endothelial cell types to evaluate the molecular pathways involved in differentiation and mineralization.
(v) For assays aimed at genotoxicity evaluation, such as the comet assay, fibroblast cell lines (e.g., L-929, C165) or primary cells (e.g., hMSCs) are used, depending on the specific objectives of the study.

Overall, while standardized cell lines offer consistency and comparability, the increasing use of human-derived cells reflects a growing emphasis on physiological relevance in biomaterials research. In many of the studies discussed later in this review, standardized cell lines are not always employed; instead, other cell types, such as human osteoblast-like cell lines (e.g., MG-63, Saos-2) or primary hMSCs, are used to more accurately simulate the biological response at the bone–material interface.

## 7. Cytotoxicity and Viability Assays

### 7.1. MTT Assay

The MTT (Methyl Thyazolyl Tetrazolium) assay is one of the most commonly used tests for evaluating cellular viability [117,118,119,120], standardized according to ISO 10993-5 [110,111]. MTT is used, for example, to evaluate whether cells have successfully adhered to the material’s surface and proliferated [121].

**Figure 5:**
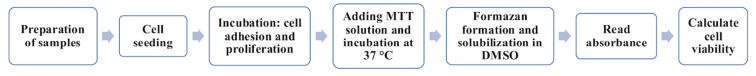
Flow chart of MTT assay.

The assay relies on the metabolic reduction in the yellow, water-soluble tetrazolium salt 3-(4,5-dimethylthiazol-2-yl)-2,5-diphenyltetrazoliumbromid into blue-purple, insoluble formazan crystals [117]. This process occurs in several steps (Figure 5) and is mediated by mitochondrial enzymes in metabolically active cells [122]. The resulting colored solution is quantified using a spectrophotometer [123,124], as the color becomes more intense with more viable cells.

Because formazan crystals are insoluble in the culture medium and accumulate inside the cells, the assay requires a solubilization step—including cell lysis—to dissolve the crystals and allow their quantification. Various solvents can be used, such as propanol, ethanol, isopropanol, or dimethyl sulphoxide (DMSO), each of which influence the outcome of the MTT assay [125]. The absorbance of MTT formazan in DMSO increases linearly with concentration, while propanol cause precipitation of proteins present in the culture medium [125].

Sylvester [126] reviewed multiple studies aimed on optimizing the MTT assay and outlined key factors influencing test response and the quantification of cell number: (i) standardizing the MTT concentration to 1 mg/mL yields reliable and cost-effective results; and (ii) a standardized incubation time of 4 h offers the optimal specific activity within a reasonable timeframe.

However, several studies have shown that incubation time is not always directly proportional to the cellular response. For example, Deb et al. [117] used the MTT assay to assess potential leachable components from porous scaffolds fabricated by mixing 45S5 Bioglass^®^ powder (45–90 μm) and polyvinyl alcohol (PVA), using a primary human osteoblast (HOB) cell line. They found that the increased alkalinity of the eluate due to longer elution times significantly reduced cell response. Nevertheless, this effect is unlikely to cause significant toxicity in vivo due to dilution across the body’s surface area. On the other hand, Zhang et al. [118] investigated the adhesion and proliferation of MSCs on A-W porous scaffolds. In this case, the MTT test showed that cells proliferated on the 3D structure over time, indicating in vitro biocompatibility. In vivo tests confirmed that the scaffold pre-incubated with cells improved bone regeneration efficiency.

Additionally, the physical form of the sample can influence the MTT response. Kaczmarek et al. [124] compared Ti-45S5 nanocomposites in disk scaffold and powder forms, observing differences in the proliferation of human fibroblast CCD-39LU cells. Specifically, the nanocrystalline powder exhibited good cytocompatibility and supported higher levels of cell growth compared to the microcrystalline disk scaffold.

In conclusion, optimizing both MTT concentration and exposure time is essential, since different cell lines showed variability in dose and time response. As demonstrated in the studies above, results can change significantly based on factors such as material form, potentially leading to false negatives and inconsistent interpretations of biocompatibility. For this reason, a multi-parametric approach [117,127] is often recommended for more accurate interpretation of MTT assay results.

### 7.2. XTT Assay

The XTT assay is a colorimetric method [114], standardized under ISO 10993-5 [107,108] and closely related to the MTT assay, as both use the reduction in tetrazolium salts through mitochondrial dehydrogenase enzymes activity in metabolically active cells. In this assay, the yellow tetrazolium salt XTT (2,3-bis(2-methoxy-4-nitro-5-sulfophenyl)-5-[(phenylamino)carbonyl]-2H-tetrazolium hydroxide) is reduced to an orange, water-soluble formazan [115]. 

**Figure 6:**
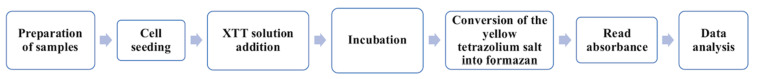
Flow chart of XTT assay.

To perform this test on sample extracts—its most common application—cells are seeded in plates containing the eluates and incubated. The XTT solution is then added, followed by a second incubation period during which the formazan is produced. The intensity of the orange product, that reflects the number of viable cells, is then measured using a spectrophotometer [115,128] (Figure 6).

The XTT assay has been applied in multi-parametric studies [114,115] demonstrating its compatibility with various cell types [129] and its sensitivity to factors such as sample form and porosity. For example,

(i) Mitri et al. [130] evaluated the viability of MSCs exposed to extracts from dense and porous HAp/β-TCP ceramic granules using a multi-assay approach combining XTT, Neutral Red, and Crystal Violet tests. The authors found that ceramic extracts significantly enhanced mitochondrial dehydrogenase activity without inducing cytotoxicity. However, in earlier studies, Mitri et al. reported cytotoxic effects from porous ceramic extracts, which were attributed to the possible release of microparticles during sintering. The observed loss in cell viability was associated with growth inhibition caused by BGs when cells were cultured in the presence of varying sample concentrations.
(ii) Deliormanli [131] used the XTT assay to evaluate the osteoblast cell response to silicate-based 13–93 BG fibers (54.6% SiO_2_, 22.1% CaO, 7.9% K_2_O, 7.7% MgO, 6.0% Na_2_O, and 1.7% P_2_O_5_) fabricated via sol–gel processing and electrospinning. The assay revealed no cytotoxicity of the scaffolds but indicated that cells may penetrate the porous structure of the biomaterial. This indicates that spectrophotometric quantification may be influenced by cell infiltration into the porous structure of the tested material, potentially leading to false negative results or underestimation of cell viability.

Beyond porosity, the XTT assay may be less sensitive in detecting very low levels of cell activity and can be affected by the presence of interfering substances in the culture environment. Funk et al. [132] demonstrated that certain components of cell culture media, such as human or bovine serum albumin, can reduce tetrazolium salts, thereby interfering with cell viability assessments. These proteins induce a concentration-dependent increase in signal, and this non-cellular reductive activity may lead to false-positive results, causing an overestimation of living cells and a reduced estimate of cytotoxic effects. Similar effects were also observed with the MTT assay. The authors showed that this interference was due to the free cysteine residue (HS-CH_2_-CH(NH_2_)-COOH) in albumin, which can be chemically bound to an organic compound to minimize assay interference.

Unlike the MTT assay, which produces insoluble formazan crystals that require cell lysis and a solubilization step, XTT produces the water-soluble formazan directly in the culture medium. This key difference makes the XTT assay simpler and faster, eliminating the need for additional processing steps, although most cells do not metabolize the salt efficiently [133]. Despite this limitation, XTT remains a valuable assay for evaluating cytotoxicity. However, the relationship between XTT concentration and the resulting spectrophotometric signal is not always linear. Therefore, accurate quantification requires generating specific standard curves for each concentration of tetrazolium salt used.

### 7.3. Alamar Blue^®^ Assay

**Figure 7:**
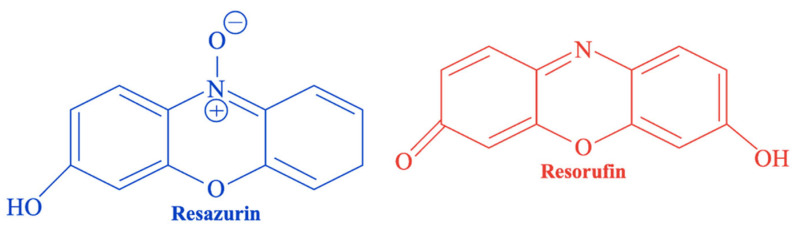
Chemical structures of resazurin (blue) and its reduced form resorufin (red) used in the Alamar Blue^®^ assay.

Alamar Blue^®^ is a resazurin-based, ready-to-use assay, commonly used to assess cell proliferation and viability. It relies on the reduction in resazurin—a blue, non-fluorescent, water-soluble, and cell-permeable redox indicator—into resorufin, a red and fluorescent compound (Figure 7) [134,135]. This conversion occurs only in metabolically active cells and is mediated by mitochondrial enzymes [136]. The resulting fluorescence intensity directly reflects cell viability. Resorufin (i) enhances the fluorescence and color of the surrounding medium, and (ii) enables viability assessment without the need for cell lysis [134], unlike the MTT.

**Figure 8:**
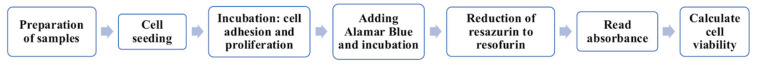
Flow chart of Alamar Blue^®^ assay.

To perform the test (Figure 8), the culture medium in all wells containing the sample extracts is replaced with the Alamar Blue^®^ solution and incubated. Following the reduction in resazurin, fluorescence is measured. Results are typically expressed as the percentage of Alamar Blue^®^ reduction. Cell proliferation increases metabolic activity, leading to more reducing conditions in the culture medium; conversely, growth inhibition results in a more oxidizing environment [117,137].

Several studies have shown that the accuracy of the Alamar Blue^®^ assay can be influenced by the structural properties of the tested biomaterials: for example, (i) Chen et al. [137] investigated osteoblast-like (MG 63) cells’ proliferation on macro- and micro-structured 45S5-derived glass-ceramic scaffolds; (ii) Vasconcelos et al. [131] analyzed nanostructured PLA-45S5-carbon nanotubes scaffolds; (iii) Longhin et al. [138] assessed the cytotoxicity of nanostructured biomaterials; and (iv) Ibrahim et al. [139] evaluated the application of the assay on synthetic HAp and β-TCP powder extracts. These studies demonstrated that the microporous structure promoted cell infiltration and extracellular matrix formation, resulting in increased fluorescence intensity. Conversely, in the nanostructured samples, no significant changes in cell viability were observed between different inner spacings, suggesting that nanoscale modifications may have affected metabolic activity rather than directly influencing proliferation. Moreover, fluorescence-quenching effects caused by material absorption, as well as possible chemical interaction between nanomaterials and Resazurin, may lead to underestimation of cell viability (false negatives).

Reductases responsible for Resazurin reduction are located not only in the mitochondria but also in other subcellular compartments. This can affect data interpretation, potentially leading to over- or underestimation of cell response [140]. Consequently, the correlation between Resazurin reduction and cell proliferation is not always linear and may vary depending on cell type, growth phase, and experimental conditions [140]. Unlike MTT and XTT, Alamar Blue^®^ allows for real-time monitoring of cell viability, making it suitable for long-term studies and the detection of low levels of cytotoxicity.

### 7.4. Neutral Red Uptake (NR) Assay

Neutral Red (NR) is a widely used standardized assay [110,111] for evaluating cytotoxic effects of materials and quantify the number of viable cells in a culture [114,141]. The test relies on the ability of living cells to accumulate the weak cationic dye Neutral Red within their lysosomes. Cellular uptake of the dye enables distinction between viable, damaged, and dead cells. Cytotoxicity is expressed as a reduction in NR uptake after exposure to the test material. It should be noted that (i) alterations of the lysosomal membrane or changes to the cell surface reduce NR accumulation, leading to lower optical density readings during spectrophotometric analysis; and (ii) dead or severally damaged cells may lose membrane integrity, which can result in non-selective dye accumulation. This interaction can sometimes result in elevated signals, potentially compromising result interpretation.

**Figure 9:**
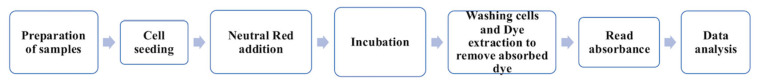
Flow chart of NR assay.

To perform the test using a direct contact approach—one the most commonly used methodologies [79,141]—cells are first cultured, and the medium is replaced with an NR solution. After incubation, the dye is removed from the cells using an ethanol/acetic acid solution to form a homogeneous mixture. The test is usually performed in triplicate, and NR uptake is quantified using spectrophotometry [109,115] (Figure 9).

However, as reported in several studies, including that by Bellucci et al. [78], the NR direct contact test can produce different results compared to assays on sample extracts. In their work, various HAp-BG composites in disk form were evaluated. While all samples appeared non-cytotoxic in the direct NR assay using murine fibroblasts, the biological responsiveness assessed by the BrdU test—a proliferation assay based on the incorporation of bromodeoxyuridine (BrdU) into newly synthesized DNA—was significantly lower when performed on the corresponding extracts. Similarly, Ciapetti et al. [142] evaluated cytotoxicity using different methods, including the NR test. Depending on the assay, cells were exposed to material extracts for different durations. According to these studies, discrepancies between results may be attributed to: (i) the possible release of particulates from the samples, which can affect cell viability and absorbance measurements [78]; (ii) the differing sensitivity of the assays [142]; or (iii) prolonged exposure times leading to overestimation of cell viability.

However, not all studies report such differences. For instance, Sergi et al. [109] investigated fibroblast cell viability and proliferation on chitosan/BG wound dressings and demonstrated that biological assays using different approaches can yield comparable results. In their work, biological responsiveness was evaluated using both the NR direct contact test and the MTT assay on extracts to minimize interference from particulates released by the materials. Their findings suggest that (i) the material processing, from grinding to sintering, does not compromise biological evaluation; and (ii) the material composition plays a critical role. Specifically, the release of ions, such as Sr, Mg, and Zn appears to support specific cellular responses through activation of signals involved in the cell cycle.

In conclusion, although the NR test can sometimes yield different results compared to other assays, it remains a useful and sensitive method for assessing cell viability. When combined with complementary techniques, it can provide valuable insights into the biological responsiveness of biomaterials. However, since the literature reports conflicting outcomes, further studies are needed to clarify and better understand the reliability of NR in different contexts.

The main advantage of the NR assay lies in its simplicity for evaluating cellular health and proliferation, requiring minimal preparation and processing. However, a limitation of the NR assay is that dead or severely damaged cells may non-selectively accumulate the NR due to compromised membrane integrity. Without intact membranes and the lysosomal pH gradient, these cells can not regulate dye uptake, leading to passive accumulation and potential overestimation of cell viability (false positives). Moreover, certain compounds, such as degradation products, may interact with the dye or interfere with the assay system, affecting the accuracy of the results.

### 7.5. Trypan Blue Exclusion Test

The Trypan Blue exclusion test is commonly employed to determine the number of viable cells in a cell suspension [127]. This method relies on the principle that viable cells with intact membranes exclude the polar dye Trypan Blue, whereas in dead cells with compromised membranes, the dye can penetrate and stain the cytoplasm blue [127].

**Figure 10:**
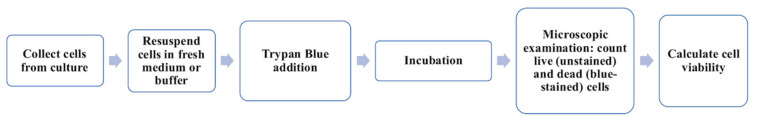
Flow chart of Trypan Blue exclusion test.

The cell suspension is mixed with the dye and then observed under a microscope to count the number of viable and dead cells (Figure 10). Strober [116] proposed calculating the percentage of viable cells using the following Equation (2):

(2) $$Viable\;cells\;\left(\%\right)=\left(\frac{Total\;number\;of\;viable\;cells\;per\;mL\;of\;aliquot}{Total\;number\;of\;cells\;per\;mL\;of\;aliquot}\right)\cdot 100$$

Therefore, the cell viability is assessed indirectly through membrane integrity, which may limit accuracy of the results [116]. For instance, cells may appear viable in this test even during the early stages of cell death, such as apoptosis, or under conditions of metabolic stress. These cells can still exclude the dye. Conversely, cells might take up the dye due to slight membrane damage, even though they are still viable. This can lead to misinterpretation of the results, with false negatives or false positives (i.e., viable cells counted as dead) [143]. However, no quantitative studies specifically demonstrating these limitations in the context of BGs or ceramics are currently available.

Another important aspect influencing the test outcome is the porosity of the material [144]. Open and interconnected pores can retain dye, potentially interfering with microscopic interpretation and leading to false positives. Moreover, high porosity (e.g., macropores) facilitate oxygen diffusion, thereby supporting cell viability. A relevant example was proposed in the study by Vitale-Brovarone et al. [144], where the Trypan Blue assay was used to assess the viability of human osteoblast cells on macroporous glass-ceramic scaffolds produced with a polyurethane sponge template and BG powders. This study illustrates the complexity of interpreting Trypan Blue results in porous systems, where porosity may influence dye distribution and retention, possibly complicating the analysis. For these reasons, the Trypan Blue assay should be combined with additional viability tests as part of a multi-parametric evaluation approach.

### 7.6. Crystal Violet Staining

Crystal Violet staining is a versatile assay commonly used to evaluate cell adhesion and indirectly assess cell viability [145], based on the dye’s ability to bind to cellular proteins and other negatively charged components [115]. This method is based on the capacity of the basic dye to bond with negatively charged molecules and acidic groups within the cells and the extracellular environment [146]. In viability assays, the retained quantity of Crystal Violet is proportional to the number of adherent cells, allowing for the quantification of cell density and growth [147].

**Figure 11:**
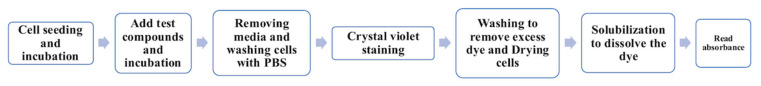
Flow chart of Crystal Violet staining assay.

To perform the test using a direct contact approach (Figure 11), cells are first allowed to firmly attach to the surface of the culture plates. The Crystal Violet staining solution is then added to each well. After incubation, the plate is washed four times with water and left to air-dry. Subsequently, methanol is added to each well to solubilize the bound dye, and the optical density (OD) is measured. The average OD of the non-stimulated control is set as the reference value, corresponding to 100% [147].

Crystal violet remains an important tool for assessing cell viability; however, the assay may also stain dead cells that remain attached to the plate, potentially resulting in false positives. An example of this limitation was reported by Tavares et al. [148], who evaluated the cytocompatibility of β-TCP and Mg-substituted β-TCP (β-TCMP) granules (250–500 μm) using pre-osteoblast cells. Among the assays employed, the Crystal violet test showed considerable cell density even after 1% phenol treatment, a cytotoxic agent known to induce complete cell death. In contrast, the XTT assay detected no viable cells under the same conditions. Based on these findings, the authors concluded that Crystal violet can penetrate dead cells adhered to the culture plate, leading to an overestimation of cell viability and false-positive results.

However, this issue is not consistently observed across all studies. For instance, Pires et al. [149] demonstrated in their work on HAp blocks that Crystal violet staining correlated with other metabolic assays, such as the MTT. Their results suggest that Crystal violet can still provide reliable information on viable cells under certain experimental setups, without leading to false positives. These contrasting results highlight the importance of combining Crystal Violet with complementary assays to ensure an accurate assessment of cell viability. Moreover, the test requires microscopic analysis, making it more labor-intensive and less quantitative than spectrophotometric assays; the fixation and staining steps may alter cell morphology, and the assessment can be affected by various interferences.

Consistently with the Trypan Blue staining, surface roughness and topography can significantly influence both cell adhesion and the results of Crystal Violet staining. When a material supports good cell attachment and proliferation, the resulting coloration is more intense and homogeneous. Nevertheless, several studies [150,151] reported no cytotoxic effects or correlation between particle characteristics and staining when using materials with varying surface areas and pore sizes. This suggests that Crystal Violet staining can still be a useful method for assessing cellular behavior, if its known limitations are carefully considered.

## 8. Proliferation Assay

### 8.1. Bromodeoxyuridine (BrdU) Assay

**Figure 12:**
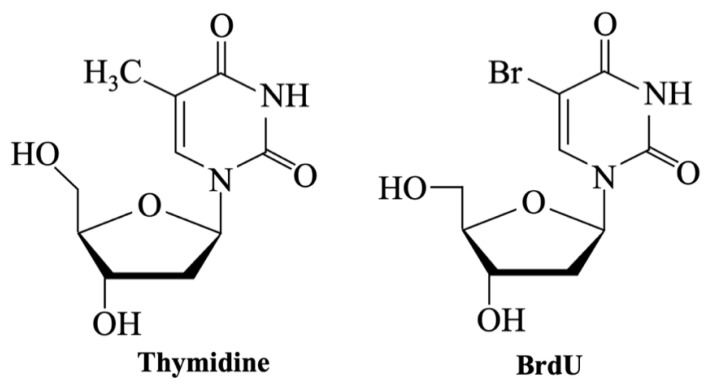
Chemical structures of Thymidine and BrdU.

The BrdU assay is a widely used method to assess cell proliferation [78,152] that measures the incorporation of 5-bromo-2′-deoxyuridine into the DNA of proliferating cells [18,153]. BrdU is structurally similar to thymidine [154] (Figure 12) and is incorporated into actively dividing cells during the S-phase of the cell cycle [155], but it inserts a bromine atom into DNA, potentially altering normal function and causing toxicity [156].

**Figure 13:**
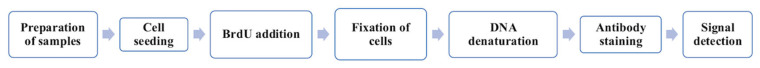
Flow chart of BrdU assay.

In this assay (Figure 13), cells are cultured with biomaterial extracts in multi-well plates for a defined period—usually 24–48 h. After incubation, a BrdU labeling solution is added to the culture medium, allowing incorporation into the DNA of actively proliferating cells. Following the labeling period, cells are fixed, and their DNA is denatured to enable detection of the incorporated BrdU. A BrdU-specific antibody is then applied and binds to the labeled DNA. The resulting immune complexes are subsequently detected through a colorimetric reaction, and absorbance is measured at an appropriate wavelength. The signal intensity is directly proportional to the amount of newly synthesized DNA, providing a quantitative measure of cell proliferation [157].

The BrdU assay is highly sensitive and adaptable to different cell types, but it has some limitations: detection requires harsh treatments, such as DNA denaturation using HCl, enzymatic digestion (e.g., trypsin), or heat—which may render the samples incompatible with other staining techniques [158]; moreover, the results of BrdU assay can vary significantly depending on factors like cell type, culture conditions, and the concentration of bioactive compounds. Notably, the literature presents inconsistent findings regarding how these variables influence BrdU incorporation. For example:

(a) The study by Bielby et al. [159] highlights this critical limitation. The authors used BrdU to assess the proliferation of primary murine and human osteoblasts in response to the dissolution products of 58S BG (60% SiO_2_, 36% CaO, 4% P_2_O_5_). Murine osteoblasts showed a 100% increase in BrdU uptake by day 6, indicating a strong mitogenic response. In contrast, human osteoblasts exhibited an early inhibition of BrdU incorporation (day 2), followed by stimulation (day 4), and then inhibition again (day 6). These findings suggest that the soluble ions released from the glass can modulate cell proliferation, but the response is highly cell-type dependent. The inhibitory effects observed at higher glass concentrations were likely due to increased medium pH and cytotoxicity from elevated cation levels, which were not present at lower concentrations.
(b) While Bielby et al. [159], reported a dose-dependent inhibition of BrdU incorporation at high concentrations of undoped 58S BG, other studies suggest that modifying the glass composition can mitigate these adverse effects. For instance, Malavasi et al. [154] investigated Ce-doped BGs based on the 45S5 composition using long-bone osteocyte-like (MLO-Y4) and fibroblast (NIH/3T3) cell lines, and observed no cytotoxicity or inhibition of proliferation, even at elevated BG concentrations. On the contrary, BrdU incorporation increased with the cerium content in the glasses.

A further limitation is that BrdU detects DNA synthesis during the S-phase of the cell cycle but cannot determine whether cells actually complete mitosis or remain viable, potentially leading to false-positive results. Silver et al. [160] provided key insights into this limitation. Their study revealed that cells can incorporate BrdU even in the absence of cell division, particularly under metabolic stress. They observed that exposure to 45S5 Bioglass^®^ increased intracellular pH and lactate production in osteoblasts, indicating elevated metabolic activity. However, this did not always correlate with higher BrdU uptake, suggesting that the assay may reflect DNA synthesis associated with stress responses rather than true proliferation. For this reason, Silver et al. recommended combining BrdU with complementary assays, such as MTT for cell viability and alkaline phosphatase activity for differentiation.

### 8.2. 5-Ethynyl-2′-deoxyuridine (EdU) Assay

**Figure 14:**
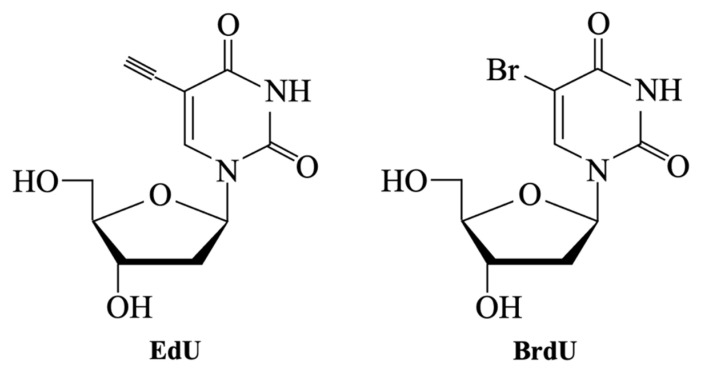
Chemical structures of EdU and BrdU.

In 2008, Salic and Mitchison [161] developed a novel assay based on the incorporation of 5-ethynyl-2′-deoxyuridine (EdU—Figure 14) into DNA. Incorporated EdU is detected via a copper-catalyzed cycloaddition (“click” chemistry) between its ethynyl group and a fluorescent azide [162]. This assay labels newly synthesized DNA and allows direct detection of the thymidine analog within cells [158], without requiring DNA denaturation.

Zeng et al. [162] demonstrated that the number of EdU-stained cells increased in a dose-dependent manner. Moreover, the fluorescent intensity of labeled cells amplified as the EdU doses increased. The authors also proposed an Equation (3) to calculate the number of EdU-positive cells (*N*):

(3) $$\;N=\frac{D\cdot N_{max}}{D50+D}\cdot 100$$

where *D* is the EdU dose, *N_max_* is the maximum number of EdU-positive cells, and *D*50 is the EdU dose at which the number of EdU-positive cells equals half of *N_max_*.

Subsequently, the authors compared the results with the standard BrdU technique, and both labeling assays yielded a comparable number of proliferating cells. These findings indicate that EdU is as sensitive as the BrdU assay.

Compared with BrdU, EdU provides notable practical advantages that enhance the reliability of proliferation studies, especially with complex samples such as BGs or ceramic biomaterials [163]. EdU does not require DNA denaturation, it minimizes morphological alterations and preserves nuclear and cellular integrity, making the assay faster and more reliable [158]. Additionally, EdU offers high sensitivity and maintains the physical integrity of samples, making it a valid and more convenient alternative to BrdU, or a complementary method that can be used in conjunction with it [162].

Nevertheless, although EdU is increasingly popular in proliferation studies, the literature still lacks specific investigations into its limitations in the context of BGs or ceramics. Most available data focus on comparisons with BrdU in general cell culture settings [158,164]. For this reason, dedicated studies are needed to fully understand the potential and limitations of EdU in biomaterials research.

## 9. Differentiation Assays

### 9.1. Alizarin Red Staining

Alizarin Red staining is a widely used technique for evaluating the effects of biochemical and morphological signals on in vitro osteogenic differentiation [165,166,167]. The assay is often regarded as the gold standard for detecting and quantifying matrix mineralization by osteoblasts [168]. Alizarin Red can also serve as a useful initial screening tool to assess the biocompatibility and the ability to promote mineralization of calcium-containing biomaterials, offering a complementary alternative to animal testing [169].

**Figure 15:**
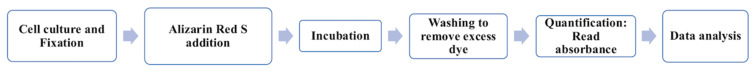
Flow chart of Alizarin Red staining assay.

The dye specifically reacts with Ca^2+^ ions via a chelation process [168]. It is water-soluble and contains catechol functional groups, which can also interact with metal oxide surfaces [170,171]. The procedure involves fixing the cell cultures and incubating them with Alizarin Red solution at a slightly acidic pH to facilitate calcium binding. Following washing to remove excess dye, calcium-rich deposits become visible as red-orange. They can be evaluated qualitatively under a microscope or quantified by extracting the bound dye and measuring its absorbance using a spectrophotometer (Figure 15).

An important consideration when interpreting Alizarin Red results is the potential influence of ions released from biomaterials on cellular behavior. Many BGs and ceramics gradually release ionic species, including Ca^2+^, Mg^2+^, Zn^2+^, phosphate (PO_4_^3−^), and silicate (SiO_4_^4−^) ions, which can modulate both cell proliferation and osteogenic signaling cascades. These ions may indirectly affect mineral deposition by altering the cellular microenvironment, leading to over- or underestimation of osteogenic activity. Consequently, Alizarin Red staining may not always accurately reflect true matrix mineralization, especially in the presence of highly reactive or ion-leaching materials. Some case studies have illustrated these limitations and emphasized the importance of integrating this assay with complementary analytical techniques. For example:

(i) Zhao et al. [167] employed the Alizarin Red staining technique to evaluate the mineralization potential of bone marrow stromal cells (BMSCs) cultured with extracts from 45S5, β-TCP, and a phosphate-rich bioactive glass containing no Na_2_O (PSC). Their results showed that PSC significantly enhanced calcium deposition compared to the other materials, as evidenced by more intense Alizarin Red staining and higher absorbance values. The relatively stable pH environment resulting from the release of silicate, calcium, and phosphate ions from PSC supported improved cell viability and mineralization and contributed to the enhanced proliferation of BMSCs.
(ii) The results reported by Vargas et al. [169] support the findings of Zhao et al. [167] regarding both the effectiveness of Alizarin Red staining and the influence of released ions on mineralization. Additionally, Vargas et al. proposed a dual-staining method combining Alizarin Red with Alcian blue—a polyvalent basic dye—for a more comprehensive evaluation of the biological responsiveness [169]. This approach may help mitigate the risk of false-positive results. Alizarin Red may also bind non-specifically to calcium-containing residues [168], such as precipitated calcium phosphate derived from the culture medium or degradation by-products of BG. This non-specific binding may lead to an overestimation of osteogenic differentiation. The inclusion of Alcian Blue, which stains sulfated glycosaminoglycans, aids in distinguishing genuine mineralization from such artifacts.

Furthermore, the assay exhibits only moderate sensitivity, highlighting the need for optimization of the staining procedure. Bernar et al. [168] suggested adding calcium chloride to the culture medium to promote cell differentiation and bone mineralization in a time- and cost-effective manner. However, other treatments, such as the addition of calcitonin or increasing the surface area, did not enhance the sensitivity of the Alizarin Red assay.

### 9.2. Alkaline Phosphatase (ALP) Activity Assay

The alkaline phosphatase (ALP) activity assay is a commonly used method for evaluating osteoblastic differentiation and early-stage bone formation. ALP is an enzyme essential for initiating matrix mineralization and serves as an early indicator of osteogenesis [172].

**Figure 16:**
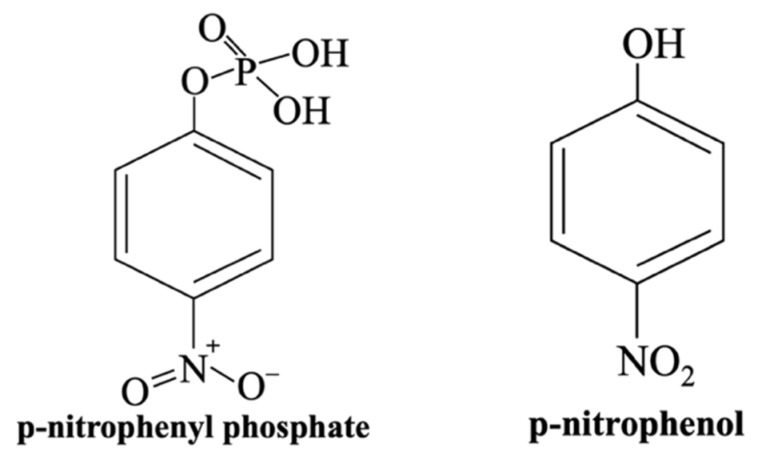
Chemical structures of p-nitrophenyl phosphate and p-nitrophenol, as converted by the ALP activity assay kit.

**Figure 17:**
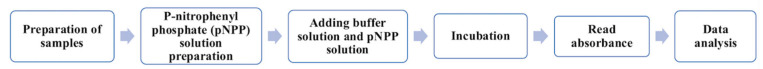
Flow chart of ALP activity assay.

The assay involves cultured osteogenic cells, such as MSCs or osteoblast-like cells, and measures enzyme activity using an ALP assay kit [121,123,173]. The kit catalyzes the hydrolysis of p-nitrophenyl phosphate into p-nitrophenol (Figure 16), producing a colorimetric or fluorescent signal proportional to ALP activity. By continuously measuring absorbance at 405 nm, the enzyme activity can be quantified [166,172,173] (Figure 17). Results are typically expressed as specific enzyme activity, reported in nmol/min/mg [172].

The release of bioactive ions, including SiO_4_^4−^ and Na^+^, from biomaterials contributes to matrix mineralization and cell maturation. Several studies in the literature support the ion-dependence of ALP activity [121,122]. The research conducted by Hesaraki et al. [122] explored this concept by comparing the ALP activity of osteoblastic cells cultured on pure HAp and on a composite material consisting of HAp reinforced with Sr-containing BG (Sr-BG) nanoparticles. In their study, osteoblastic cells were directly seeded onto the surface of sintered samples (direct contact approach). Although cell proliferation was similar between the two materials, ALP activity was significantly higher in the HAp-SrBG composite. This enhancement was attributed to the release of Sr^2+^ and SiO_4_^4−^ ions into the culture medium, which are known to promote osteogenic differentiation. Moreover: (i) the concentrations of Sr and Si increased with both the BG content and culture time; (ii) on day 14, a decline in ALP activity was observed in the HAp-SrBG samples, potentially indicating a transition from the early differentiation stage to the mineralization phase.

Similarly, Spirandeli et al. [121] investigated ALP activity in human osteoblastic MG-63 cells directly cultured on β-TCP scaffolds reinforced with sol–gel 45S5 Bioglass^®^. Their findings revealed a marked increase in ALP activity for the composite scaffolds, supporting that biological responsiveness is strongly dependent on the release of bioactive ions.

In addition to the demonstrated relationship between ion release and ALP activity, also BG processing differences may affect the biological response [174]. Surface charge and particle size correlate with ALP expression, even if results in the literature are not always consistent. El-Sayed et al. [174] compared the influence of the synthesis on the in vitro biological responsiveness of two quaternary BGs. Their results suggest that (i) both SG and MQ enhance ALP activity, although SG led to a higher ALP response; (ii) SG promoted the formation of multilayered cells, favoring cell–cell adhesion. Conversely, Fonseca et al. [123] evaluated the ALP activity of MSCs seeded on electrospun polycaprolactone (PCL) membranes combined with BG produced by either SG or MQ. After 10 days, cells cultured on BG-containing PCL membranes showed increased ALP activity compared to those on pure PCL membranes, confirming the stimulatory effect of BG-released ions on osteogenic differentiation. In this case, PCL membranes with MQ-derived BGs induced higher ALP levels than those with sol–gel-derived BGs. Therefore, production methods represent a critical factor that should be carefully considered and further investigated to fully understand their impact on biological performance. 

In conclusion, conventional methods for ALP detection often lack sufficient sensitivity, precision, and versatility across a wide range of sample types [175]. These limitations become relevant when working in presence of BG degradation products or interfering ions, which may affect absorbance measurements, leading to under- (false negative) or overestimation (false positive) of ALP activity. Consequently, several studies have proposed improved ALP detection strategies. Various analytical approaches have been explored, including optical techniques such as fluorescence and chemiluminescence, which offer higher sensitivity, as well as electrochemical and electrophoretic methods, which allow real-time monitoring and better discrimination in complex environments.

## 10. Gene and Protein Expression Assays

### 10.1. Quantitative Polymerase Chain Reaction (qPCR)

**Figure 18:**
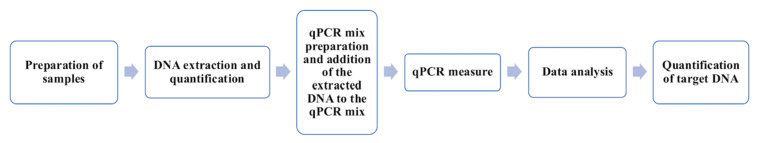
Flow chart of qPCR assay.

Quantitative PCR (qPCR) is a technique for measuring gene expression associated with cellular differentiation [176,177,178], and for quantifying mRNA levels of key differentiation markers, including osteocalcin [166] and various types of collagens [179]. The method involves the real-time amplification of targeted gene sequences, where fluorescence emitted by a DNA-binding dye provides quantitative information on gene expression [180] (Figure 18).

The use of qPCR in bone TE has been demonstrated in numerous studies, showing that mRNA expression and corresponding protein levels increase in a dose-dependent manner in response to the amount of BG or dopants in the biomaterial.

(i) Mosaddada et al. [166] used qPCR to analyze the osteogenic differentiation of cells seeded on collagen/Sr-doped BG scaffolds. Increasing Sr content led to an upregulation of gene and protein expression levels, which correlated with enhanced mineralization and bone regeneration.
(ii) Zhang et al. [181] employed real-time qPCR (RT-qPCR) to investigate the osteogenic potential of BG-ceramic (BGC) coatings. PCR analysis was performed on three experimental samples, with results reported as target gene expression levels. These findings confirmed a dose-dependent significant upregulation of key osteogenic genes, including ALP, osteocalcin, and Runx2, in bone marrow MSCs cultured on BG-coatings. Higher BG content induced stronger gene expression responses.
(iii) Furthermore, Morales-Hernandez et al. [177] applied qPCR to assess osteogenic gene expression in normal human osteoblasts (NHOsts) cultured on macroporous composite scaffolds composed of PLG and different bioceramics (HAp, TCP, and 45S5 Bioglass^®^). Their results demonstrated that BG-containing scaffolds significantly up-regulated the expression of bone matrix proteins, such as COL1A1 and SPARC, compared to HAp- and TCP-based composites, supporting the enhanced osteogenic potential induced by BG. BGs degrade more rapidly than HAp and TCP, triggering cellular processes through the release of ionic degradation products, such as silica ions. Overall, these observations also reinforce the concept that composite systems with BGs or bioceramics represent a highly promising class of materials, which can be engineered to enhance biological responsiveness [182,183].

In conclusion, qPCR is a powerful technique for assessing gene expression in biomaterials research, although it presents both advantages and limitations: (i) real-time qPCR uses purified DNA or RNA as test samples, and the stability of nucleic acids means they should not be evaluated according to the same assay guidelines as other samples types [184]; (ii) the accuracy of qPCR can be affected by several factors, including RNA quality, PCR efficiency, and the choice of reference genes; (iii) qPCR can detect and quantify very low levels of mRNA with high specificity; (iv) results can be obtained within a few hours; (v) qPCR requires only small amounts of RNA, which is useful when working with limited cell numbers.

### 10.2. Immunocytochemistry

**Figure 19:**
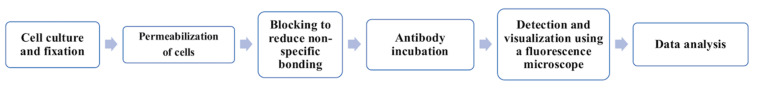
Flow chart of Immunocytochemistry technique.

Immunocytochemistry is a sophisticated technique for biological evaluation, allowing the visualization and precise localization of specific proteins within cells via antibody-based fluorescent labeling [185]. The procedure involves cell fixation and permeabilization [185,186], followed by incubation with primary antibodies and detection with fluorescently labeled secondary antibodies (Figure 19) [187,188,189].

Some studies have demonstrated immunocytochemistry potential for evaluating cell–material interactions. Regarding structural properties, in contrast to other techniques previously described, material porosity does not drastically alter cell adhesion, as shown by Usseglio et al. [190] They applied immunocytochemistry to evaluate endothelial cell responses to microporous HAp-based ceramics. The analysis confirmed that cells adhered to both dense and porous HAp surfaces, without significant differences in the distribution of focal adhesion markers.

Cell behavior may instead be influenced by the presence of dopants in BGs and by their resulting microstructure, as illustrated by:

(i) Trandas et al. [185], who investigated Eu- and Ag-doped BG thin films. Using immunocytochemistry, they visualized cytoskeletal organization—particularly actin filaments—in pre-osteoblasts.
(ii) Similarly, Lavric et al. [186] compared BG thin films doped with Ag and Sm, produced via pulse laser deposition (PLD) and spin coating (SC). Cell adhesion and viability were evaluated using immunocytochemistry with mouse pre-osteoblasts. Their in vitro results showed that the samples supported mineralization and promoted cell adhesion and proliferation, attributed to the presence of dopant ions.

The enhanced cellular response observed on doped BG thin films can be attributed to the release of bioactive dopant ions, which modulate cellular signaling pathways, promote cytoskeletal organization, and improve protein adsorption at the material surface. These effects collectively lead to better cell adhesion, proliferation, and differentiation compared to undoped BGs or ceramics. Moreover, immunocytochemistry allows for detailed visualization of cytoskeletal organization, providing direct insights into how dopant ions influence cell-material interactions at the molecular level.

The evaluation of cytoskeletal organization and adhesion markers is critical for understanding biomaterial biological responsiveness, highlighting a key advantage of immunocytochemistry: its ability to produce high-resolution images with precise protein localization [185]. However, the method is labor-intensive, requires specialized imaging equipment, and the fixation process may introduce morphological and functional alterations in cells.

## 11. Genotoxicity Assay

### Comet Assay

**Figure 20:**
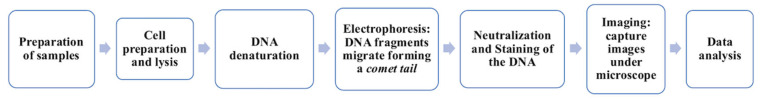
Flow chart of comet assay.

The Comet assay, also known as single-cell gel electrophoresis (SCGE), is an alkaline-sensitive technique used to assess DNA damage by detecting both single- and double-strand breaks in individual cells [191]. In this method, cells are embedded in agarose, lysed to remove membranes and proteins, and subjected to a weak electric field, causing fragmented DNA to migrate toward the anode due to its negative charge. The migrating DNA forms a comet tail, with the intact nucleoid representing the “head” and the fragmented DNA constituting the “tail”. Following electrophoresis, DNA is stained with a fluorescent dye and examined under a fluorescence microscope (Figure 20). The extent of DNA migration, quantified using parameters such as tail length and the percentage of DNA in the tail, provides insights into the degree of DNA damage in individual cells [13,191].

Several studies have shown that biomaterials with larger surface areas, such as scaffolds or nanoparticles, can enhance cell proliferation and biological responsiveness compared to materials with smaller surface areas. Kido et al. [192] investigated the genotoxicity of Biosilicate^®^ glass-ceramic scaffolds using the Comet assay on osteoblasts and fibroblasts exposed to the material over different time periods. Their analysis showed no detectable DNA damage in either cell type. Tavakoli et al. [189] used the Comet assay to study the genotoxicity of newly synthesized BG nanoparticles and Novabone^®^ BG microparticles using cultured periodontal C165 fibroblast cells. They analyzed tail length, percentage of DNA in tail, and the tail moment, calculated as the combined value of tail length and fluorescence intensity in the tail region, to quantify DNA damages. Their findings demonstrated that the novel BG nanoparticles exhibited no genotoxicity, in contrast to the Novabone^®^ microparticles. This difference was attributed to greater ion release from the nanoparticles, which is facilitated by their larger surface area, highlighting how particle size and surface properties can directly influence cell responses.

Beyond surface characteristics, dose-dependent increases in DNA damage with rising concentrations of tested biomaterials have been reported. For example, Jantová et al. [14] explored this aspect by assessing the genotoxic effects of fluor-hydroxyapatite (FHA) and fluorapatite eluates on fibroblast cells, comparing them with HAp eluates. Although the genotoxic effects were relatively mild, they progressively increased in the order HA < FHA < FA, suggesting that (i) fluoride incorporation may enhance genotoxic potential, and (ii) the release of fluoride ions may contribute to the observed DNA strand breaks.

In conclusion, the Comet assay is an effective method for investigating cellular DNA damage and repair mechanisms; however, it is complex and labor-intensive. Variability in experimental conditions, including electrophoresis settings, pH, and buffer composition, can significantly affect results, making standardization essential for data reliability [193]. Additionally, while the assay is highly sensitive, its specificity can be improved by incorporating lesion-specific enzymes that enable more precise differentiation between types of DNA damage [193]. One key limitation is its difficulty in distinguishing apoptotic cells, which are programmed to die, potentially leading to misinterpretations if appropriate controls are not implemented [193].

## 12. Comparison Between In Vitro Biological Responsiveness Tests

A variety of in vitro assays are available to evaluate biological responsiveness to biomaterials, each with distinct advantages and limitations (Table 10), which can affect result interpretation.

Trypan Blue and NR provide simple viability screening, although results may be influenced by non-specific staining or lysosomal alterations. MTT, XTT, and Alamar Blue^®^ are widely used metabolic assays; MTT requires a solubilization step, whereas XTT and Alamar Blue^®^ offer simplified protocols. The BrdU assay, commonly used to assess proliferation, requires cell fixation and DNA denaturation, limiting its application in live-cell imaging. Crystal Violet staining is effective for evaluating cell adhesion but requires microscopic analysis, making it less suitable for high-throughput screening. ALP activity assays are valuable for detecting early osteogenic differentiation but should be interpreted in conjunction with long-term differentiation markers. Advanced techniques such as qPCR, immunocytochemistry, and the Comet assay offer high sensitivity and specificity for evaluating gene expression, protein localization, and genotoxicity, respectively, although they require specialized equipment and technical expertise.

Overall, although each assay provides valuable insights into cell viability and function, method selection depends on the specific experimental objectives. The sensitivity of different cell lines also plays a critical role in evaluating the biological responsiveness to biomaterials. Therefore, combining multiple assays is often recommended to achieve a more comprehensive and reliable assessment of cellular responses.

## 13. Conclusions

The assessment of the bioactivity and biological responsiveness of BGs and ceramics presents several critical challenges, as demonstrated by the studies reviewed here. Regarding bioactivity, it can be concluded that SBF provides the most physiologically accurate ionic composition which closely approximates that of human plasma. This makes it a physiologically relevant solution, although its preparation is more complex and labor-intensive compared to TRIS or PBS. However, several studies have shown that SBF can overestimate bioactivity, producing false positives. Therefore, results should be interpreted with caution. Factors such as immersion time, the surface area-to-volume ratio, and ion concentrations can influence the extent of mineral deposition. To improve reliability, practical strategies include

- The use of revised SBF (r-SBF) with lower calcium and magnesium concentrations;
- Preventing an excessive precipitation, ensuring an adequate volume of SBF;
- Incorporating organic components to create a more realistic environment.

Nonetheless, SBF benefits from a well-established literature background, offering a wealth of detailed studies. However, results obtained using TRIS- or PBS-buffered solutions are equally valid and should be considered when evaluating bioactivity. Recently, SWF has emerged as a promising alternative for studying the bioactivity of biomaterials intended for soft tissue repair or wound healing applications. Overall, the choice of simulated physiological solution significantly influences biomaterial dissolution and bioactivity. Key factors—such as dynamic versus static conditions, ionic composition, and pH buffering capacity—must be carefully considered to ensure accurate and reproducible predictions.

Beyond bioactivity, the integration of biological responsiveness assays, partially standardized in accordance with ISO regulations, shows promise for evaluating the applicability of biomaterials. In vitro cell-based tests can be conducted using extract dilution, direct contact, or indirect contact methods, depending on the sample type and available laboratory equipment. Currently, extract-based testing is the most widely used and validated approach.

Additionally, the choice between static and dynamic culture conditions (e.g., organ-on-a-chip systems or bioreactors) significantly impacts assay outcomes, as dynamic systems better replicate physiological environments by promoting nutrient flow and mechanical stimulation. However, the use of organ-on-a-chip technology to study biological responsiveness is relatively recent, and its applications in the literature remain limited. Consequently, static culture conditions remain the most commonly employed method.

For both bioactivity and biological responsiveness, false positives and negatives remain critical challenges across methodologies, often necessitating a multiparametric approach to improve accuracy. Combining extract-based and direct contact methods, along with multiple assay types, enhances the reliability of results. Furthermore, the selection of appropriate cell lines directly influences biological outcomes and remains a key parameter. While ISO-standardized cell lines such as L-929 or BALB/c 3T3 provide reproducibility and comparability, the increasing use of human-derived cells (e.g., hMSCs or osteoblast-like cell lines) reflects a necessary shift toward more physiologically relevant models, particularly for bone tissue applications.

To improve the predictive value of in vitro assays, future research should focus on (i) refining dynamic testing setups to better mimic in vivo conditions, (ii) optimizing the composition of simulated body fluids to prevent artificial precipitation, and (iii) consistently integrating multi-parametric approaches that combine ion release analysis, structural evolution, and cellular responses. This integrative approach is crucial for guiding the development of optimized bioactive materials for tissue engineering and improving the performance of BGs and ceramics in biomedical applications.

## Figures and Tables

**Table 1:** Characteristics of static and dynamic methods for biological tests.

*Characteristic*	*Static Methods*	*Dynamic Methods*
Test conditions	Conditions are fixed and do not change during the experiment.	Conditions can vary during the experiment. - Flow rate: 0.2 mL/min has been used in some recent studies [47,48] - Fluid renewal schedules vary from complete replacement every 24 h to continuous flow.
Advantages	- Easy - Controlled conditions - More economical	- More representative of physiological conditions - Useful for studying response in dynamic environments
Disadvantages	- Do not reflect the complexity of physiological environment	- More complex to control - Require specialized equipment

**Table 2:** Ion concentrations (mM) of human blood plasma and Kokubo et al. SBF formulation (the original SBF) [53].

*Ion Concentration* (mM)	*Human Blood Plasma*	*The Original SBF*
Na^+^	142.0	142.0
K^+^	5.0	5.0
Mg^2+^	1.5	1.5
Ca^2+^	2.5	2.5
Cl^−^	103.0	148.8
HCO_3_^−^	27.0	4.2
HPO_4_^2−^	1.0	1.0
SO_4_^2−^	0.5	0

**Table 3:** Ion concentrations (mM) of revised SBFs [67].

*Ion Concentration* (mM)	*c-SBF*	*r-SBF*	*i-SBF*	*m-SBF*
Na^+^	142.0	142.0	142.0	142.0
K^+^	5.0	5.0	5.0	5.0
Mg^2+^	1.5	1.5	1.5	1.5
Ca^2+^	2.5	2.5	1.6	2.5
Cl^−^	147.8	103.0	103.0	103.0
HCO_3_^−^	4.2	27.0	27.0	4.2
HPO_4_^2−^	1.0	1.0	1.0	1.0
SO_4_^2−^	0.5	0.5	0.5	0.5

**Table 4:** Recommended ranges for SBF testing.

*Recommended Ranges*		
Mass-to-SBF volume ratio	*BGs in fine powder or particulate form*	
	1.5 mg/mL	Maçon et al. [57]
	2 mg/mL	Pirayesh et al. [59]
	1 mg/mL	Zheng et al. [62]; Bano et al. [63]
Surface area-to-SBF volume ratio	*Bulk/disk samples follow ISO 23317:2014* [78]	
	Surface area/Volume of SBF = 0.4 cm^−1^	Zhang et al. [75]; Mei et al. [69]
	Surface area/Volume of SBF = 0.05 cm^−1^	Müller et al. [68]

**Table 5:** Ion concentrations (mM) of human blood plasma and Tris-buffered solution (TRIS-HCl) [82].

*Ion Concentration* (mM)	*Human Blood Plasma*	*TRIS*
Na^+^	142.0	-
K^+^	5.0	-
Mg^2+^	1.5	-
Ca^2+^	2.5	-
Cl^−^	103.0	45.0
HCO^3−^	27.0	-
HPO_4_^2−^	1.0	-
SO_4_^2−^	0.5	-

**Table 6:** Ion concentrations (mM) of human blood plasma and PBS [82].

*Ion Concentration* (mM)	*Human Blood Plasma*	*PBS*
Na^+^	142.0	157.0
K^+^	5.0	-
Mg^2+^	1.5	-
Ca^2+^	2.5	-
Cl^−^	103.0	100.9
HCO_3_^−^	27.0	-
HPO_4_^2−^	1.0	24.9
SO_4_^2−^	0.5	-
H_2_PO_4_^−^	-	5.5

**Table 7:** Comparison of in vitro assays for bioactivity.

Solution	Main Composition	Buffering System	Advantages	Disadvantages
*SBF*	Inorganic ions, including Ca^2+^, PO_4_^3−^, Cl^−^, Na^+^	Tris-buffer	Promotes spontaneous HAp nucleation; ISO standardized for bulk samples; commonly accepted guidelines for testing BG powders	May overestimate bioactivity (false positives); sensitive to supersaturation and pH changes [46]
*TRIS*	Tris(hydroxymethyl)aminomethane, Cl^−^	TRIS-HCl	Useful for studying ion release kinetics; no interference from external Ca/P	HAp formation is not supported if the material fails to release a sufficient amount of ions [103]
*PBS*	NaCl, phosphate salts	Weak phosphate buffer	Chemically stable; suitable for long-term degradation studies [103]	Low reactivity; lacks Ca^2+^
*SWF*	NaCl, KCl. NaHCO_3_, NaH_2_PO_4_	Bicarbonate-based	Designed for wound healing materials; mimics wound environment	Not standardized; limited comparative data; rarely used for ceramics

**Table 8:** Advantages and disadvantages of in vitro methods for biological responsiveness.

	*Test on Extracts*	*Direct Methods*	*Indirect Methods*
Advantages	- Both qualitative and quantitative assessment	- Precision - Accuracy - Both qualitative and quantitative assessment	- Simplicity - Useful for preliminary studies
Disadvantages	- Influenced by several factors: area-to-volume ratio, pH, extraction duration	- Complex experimental setup	- Less representative of real biological environment - Only qualitative assessment

**Table 9:** Practical guide for selecting cell lines based on target tissue.

*Target Tissue*	*Cell Line Examples*	*Biological Parameter Assessed*
Bone	MG-63, Saos-2, hMSC	Proliferation, differentiation, ALP activity
Dentin	hMSCs	Mineralization, gene expression
Soft tissue	Fibroblasts, endothelial cells	Adhesion, proliferation, cytotoxicity

**Table 10:** Comparison of culture assays for the biological responsiveness.

Assay	Purpose	Advantages	Disadvantages	False Positives/Negatives Considerations
**Cytotoxicity and viability assays**				
*MTT* *XTT* *Alamar Blue*	Measure metabolic activity	Quantitative, user-friendly, compatible with various cell types	Potential interference from the turbidity of the material or solution	- False positives from mitochondrial hyperactivity - False negatives from non-metabolic viability
*NR*	Lysosomal integrity (viability)	Simple, no extensive preparation needed	Sensitive to variations in cell density	False positive from dye uptake by dead/dying cells
*Trypan Blue*	Cell membrane integrity	Simple, low cost	Requires manual counting; subjective	False positives if dying cells temporarily exclude the dye
*Crystal violet*	Cell adhesion and density	Simple, low cost	Requires microscopy; fixation may alter morphology	False negatives for poor staining
**Proliferation assay**				
*BrdU* *EdU*	DNA synthesis and cell proliferation	High specificity, detects active cell division	Requires cell fixation and denaturation, which may alter morphology	False negatives if cells proliferate slowly or are in quiescence
**Differentiation assays**				
*Alizarin Red*	Visualize mineralization	Direct quantification of mineral deposits	Requires fixation; may alter morphology	False negatives for poor staining
*ALP activity assay*	Osteogenic differentiation marker	Specific for osteoblastic activity; standard equipment	Time-point specific	False negatives if ALP expression has declined during later stages of differentiation
**Gene and protein expression assays**				
*qPCR*	Gene expression during differentiation	High sensitivity and specificity	Requires careful optimization	False negatives linked to dye fluorescence limitations
*Immunocytochemistry*	Protein localization and cell morphology	High-resolution imaging of cell-material interactions	Labor-intensive; requires advanced microscopy	False positives from non-specific antibody binding
**Genotoxicity assay**				
*Comet Assay*	DNA damage detection	Sensitive to various DNA lesions	Complex; labor-intensive	False positives from transient DNA breaks

## Data Availability

No new data were created or analyzed in this study. Data sharing is not applicable to this article.

## Abbreviations

The following abbreviations are used in this manuscript:

BG	Bioactive glass
TCP	Tri-calcium phosphate
A-W	Apatite-wollastonite
SBF	Simulated body fluid
TRIS	Tris-buffer
PBS	Phosphate-buffer saline solution
SWF	Simulated wound fluid
RM	Regenerative medicine
HAp	Hydroxyapatite
HCA	Carbonate hydroxyapatite
FHA	Fluor-hydroxyapatite
TE	Tissue engineering
MQ	Melt-quench technique
SG	Sol–gel process
XRD	X-Ray diffraction spectroscopy
FT-IR	Fourier-transform infrared spectroscopy
SEM	Scanning electron microscopy
OOC	Organ-on-a-chip
DCPD	Dicalcium phosphate dihydrate
CSH	Calcium sulfate hemihydrate
MTT	Methyl Thyazolyl Tetrazolium
NR	Neutral Red uptake assay
BrdU	Bromodeoxyuridine assay
EdU	5-ethynyl-2′-deoxyuridine assay
ALP	Alkaline phosphatase activity
qPCR	Quantitative Polymerase Chain Reaction
